# The Resistome of Farmed Fish Feces Contributes to the Enrichment of Antibiotic Resistance Genes in Sediments below Baltic Sea Fish Farms

**DOI:** 10.3389/fmicb.2016.02137

**Published:** 2017-01-06

**Authors:** Windi I. Muziasari, Leena K. Pitkänen, Henning Sørum, Robert D. Stedtfeld, James M. Tiedje, Marko Virta

**Affiliations:** ^1^Department of Food and Environmental Sciences, University of HelsinkiHelsinki, Finland; ^2^Department of Food Safety and Infection Biology, Norwegian University of Life SciencesOslo, Norway; ^3^Department of Civil and Environmental Engineering, Center for Microbial Ecology, Michigan State UniversityMichigan, MI, USA

**Keywords:** rainbow trout, whitefish, fish intestinal content, class 1 integrons, transposons, mobile genetic elements, qPCR array, culture-independent method

## Abstract

Our previous studies showed that particular antibiotic resistance genes (ARGs) were enriched locally in sediments below fish farms in the Northern Baltic Sea, Finland, even when the selection pressure from antibiotics was negligible. We assumed that a constant influx of farmed fish feces could be the plausible source of the ARGs enriched in the farm sediments. In the present study, we analyzed the composition of the antibiotic resistome from the intestinal contents of 20 fish from the Baltic Sea farms. We used a high-throughput method, WaferGen qPCR array with 364 primer sets to detect and quantify ARGs, mobile genetic elements (MGE), and the 16S rRNA gene. Despite a considerably wide selection of qPCR primer sets, only 28 genes were detected in the intestinal contents. The detected genes were ARGs encoding resistance to sulfonamide (*sul1*), trimethoprim (*dfrA1*), tetracycline [*tet(32), tetM, tetO, tetW*], aminoglycoside (*aadA1, aadA2*), chloramphenicol (*catA1*), and efflux-pumps resistance genes (*emrB, matA, mefA, msrA*). The detected genes also included class 1 integron-associated genes (*intI1, qacE*Δ*1*) and transposases (*tnpA*). Importantly, most of the detected genes were the same genes enriched in the farm sediments. This preliminary study suggests that feces from farmed fish contribute to the ARG enrichment in farm sediments despite the lack of contemporaneous antibiotic treatments at the farms. We observed that the intestinal contents of individual farmed fish had their own resistome compositions. Our result also showed that the total relative abundances of transposases and *tet* genes were significantly correlated (*p* = 0.001, *R*^2^ = 0.71). In addition, we analyzed the mucosal skin and gill filament resistomes of the farmed fish but only one multidrug-efflux resistance gene (*emrB*) was detected. To our knowledge, this is the first study reporting the resistome of farmed fish using a culture-independent method. Determining the possible sources of ARGs, especially mobilized ARGs, is essential for controlling the occurrence and spread of ARGs at fish farming facilities and for lowering the risk of ARG spread from the farms to surrounding environments.

## Introduction

Fish farms have been suggested as one reservoir of antibiotic resistance genes (ARGs) in the environment due to the prophylactic and therapeutic use of antibiotics (Cabello et al., 2013; Miranda et al., 2013). Because the occurrence of ARGs at fish farms leads to inefficiencies in antibiotic treatments and there is a potential risk of ARG spread from the farms to the surrounding environments, determining the sources of ARGs at fish farms is essential.

The use of antibiotics in fish farming in Finland is controlled by the Finnish Food Safety Authority (EVIRA) and requires a guideline from veterinary professionals. Oxytetracycline, a combination of sulfonamide-trimethoprim, and florfenicol are the antibiotics authorized for use in Finnish fish farms (EVIRA, 2015a,b). From 2001 to 2014, ~2.3 metric tons of sulfonamide, 0.6 metric ton of trimethoprim, 1.2 metric tons of oxytetracycline, and 0.04 metric ton of florfenicol were used in fish farming in Finland (EVIRA, 2015a,b). The antibiotics are used mainly against fish pathogens, such as *Aeromonas salmonicida, Flavobacterium psychrophilum*, and *Flavobacterium columnare*, and pathogens which occur only in sea farms, such as *Vibrio anguillarum, Yersinia ruckeri, Pseudomonas anguilliseptica*, and *Pseudomonas edwardsielloosi* (Viljamaa-Dirks, 2016).

Previously, we investigated the presence of ARGs in sediments below two fish farms in the Northern Baltic Sea, Finland (Tamminen et al., 2011a; Muziasari et al., 2014, 2016). Both farms raise rainbow trout (*Oncorhynchus mykiss*, Walbaum) and European whitefish (*Coregonus lavaretus*, Linnaeus). In the studied farms, the combination of sulfonamide-trimethoprim mixed in with fish feed was used against fish pathogens such as furunculosis-causing *A. salmonicida*. Occasionally, florfenicol was also used. Oxytetracycline had not been used since year 2000. However, records of the amount of antibiotics used at the studied farms were not available. Our previous study showed that certain ARGs encoding resistance to tetracycline, sulfonamide, trimethoprim, and aminoglycoside, an antibiotic that has never been used at the farms, were enriched in the two farm sediments even though the concentration of antibiotics in the sediments was low (1–100 ng/g of sediment). The ARGs were abundant and persistent in the farm sediments for several years but were not detected in sediments at a distance of 200 m from the fish farms. Genes associated with class 1 integrons and transposons were also the genes enriched in the farm sediments. The presence of class 1 integrons and transposons of mobile genetic elements (MGEs) in the environment can contribute to the spreading of ARGs via horizontal gene transfer systems (Aminov, 2011). Sources of the gene enrichment in the farm sediments have yet to be elucidated.

We assumed that a constant influx of farmed fish feces contributes to the enrichment of ARGs in the farm sediments. Farmed fish consume antibiotic-containing feed and selection for resistant bacteria may occur in the fish intestine during antibiotic treatment (Giraud et al., 2006). Thus, resistant bacteria carrying ARGs in the fish intestine may be secreted into fish feces and released to the surrounding water and finally to the sediments (Kümmerer, 2009). Both farms use an open cage system in which fish are kept in a net that allows free transfer of fish farming waste, containing fish feces, uneaten feed, and antibiotics, from the farms to surrounding water environments and eventually to sediments. Since the Baltic Sea has no tides and water circulation is slow (Ojaveer et al., 2010), the fish farming waste directly impacts the sediments beneath the farms.

The antibiotic resistome consists of all the existing ARGs that are capable of conferring resistance to antibiotics (Wright, 2007). Previous studies on the resistome of farmed fish detected up to 10 ARGs in cultured resistant-bacteria from skin, gills, intestinal contents, and meat (Schmidt et al., 2001; Furushita et al., 2003; Akinbowale et al., 2007; Jacobs and Chenia, 2007; Ndi and Barton, 2011). To study the antibiotic resistome in environmental samples, in which the majority of bacteria are not cultivable, culture-independent methods can be used (Perry and Wright, 2013). Highly parallel qPCR arrays provide a culture-independent method that permits the combination of hundreds of assays to detect and quantify selected known genes in a single experiment. This method has been used to analyze the resistome composition in environmental samples (Looft et al., 2012; Zhu et al., 2013; Wang et al., 2014; Su et al., 2015; Karkman et al., 2016) and fish farming environments (Muziasari et al., 2016).

In the present study, we used a highly parallel qPCR array with 364 primer sets for the detection and quantification of ARGs, other genes encoding resistance to antibacterial compounds, a mercury resistance gene, MGE associated genes, and the 16S rRNA gene. The qPCR array was used to analyze the composition of intestinal content resistomes of rainbow trout and whitefish farmed at the two Baltic Sea fish farms. The parts of farmed fish that are exposed to the surrounding water environments, such as fish skin and gills, were also analyzed to detect the possible risk of ARG spread in the fish farming environments.

## Materials and methods

### Sampling

Rainbow trout, *O. mykiss* (Walbaum), and European whitefish, *C. lavaretus* (Linnaeus) were sampled from two fish farms in the Northern Baltic Sea, Finland, in September 2014. Each farm produces ~50 metric tons of both rainbow trout and whitefish annually. The two farms used an open cage system in which each cage was 20 m in diameter and 5 m deep. The average depth was 7.5 m (±SD 1.3 m) and the average water temperature was 14.2°C (±0.85°C) during the sampling time. Detailed information of the two farms has been described previously (Pitkänen et al., 2011; Tamminen et al., 2011a,b; Muziasari et al., 2014, 2016).

Five fish from each group of small rainbow trout, big rainbow trout, small whitefish and big whitefish were sampled (Table 1). The 20 sampled fish were randomly picked from healthy fish in the net cages. The fish were bought and slaughtered using the normal fish farming process. The fish were put in boxes of ice, transported to the laboratory (duration of transport 6 h), and directly processed. The fish were individually measured and weighed. Mucosal skin and gill filaments were sampled from each fish using sterile cotton swabs before the fish abdominal cavity was opened using sterile surgical tools. The tissues and visceral fat surrounding the digestive system were removed. The portion of the digestive system consisting of the small and large intestines was removed. The content of the fish intestinal tract was gently squeezed into a zipper plastic bag and homogenized manually. The intestinal contents of each fish were analyzed individually as biological replicates. The mucosal skin and gill filaments from groups of five fish were pooled. Five fish per group were chosen to account for the minimum number of incremental samples to be taken, in line with EU commission regulation No 252/2012. The sample materials were kept at −80°C until DNA extraction.

**Table 1:** **Farmed fish samples from the Northern Baltic Sea farms**.

**Farmed fish samples**	**Rainbow trout (rt)**		**Whitefish (wf)**	
	**Small_rt**	**Big_rt**	**Small_wf**	**Big_wf**
Average weight (g)	540 (±160)	2800 (±480)	420 (±70)	780 (±100)
Time raised at the farm (months)	3	3^*^	3	15
No. of samples	5	5	5	5
History of antibiotic treatments at the Baltic Sea farms^**^	1.5% of sulfonamide-trimethoprim in fish feed at the farm in August 2014	No antibiotic treatment at the farms	No antibiotic treatment at the farms	No antibiotic treatment at the farms

* *The Baltic Sea farms also raise fish that are already mature fish*.

** *History of antibiotic treatments before entering the Baltic Sea farms was not available*.

### DNA extraction

Total DNA was extracted directly from 200 mg of each homogenized intestinal content sample using the QIAamp DNA Stool Mini Kit (Qiagen Sciences, Germantown, MD) and the pre-treatment using FastPrep method (MP Biomedicals, Irvine, CA, USA) following the manufacturer's instructions. In addition, total DNA from a swab of mucosal skin and gill filaments was extracted using the Cador Pathogen Mini Kit (Qiagen Sciences, Germantown, MD) and the pre-treatment B2 according to the manufacturer's instructions. The pre-treatment step was added in each DNA extraction method to improve the quality and yield of DNA for the qPCR array measurement. The DNA quality and concentration were analyzed with a Nanodrop 1000™ spectrophotometer (Thermo Scientific, Wilmington, DE, USA).

### The qPCR array

The qPCR reactions were performed using 364 primer sets (Table S1). Validation of the primer sets was confirmed in the previous studies (Looft et al., 2012; Pitkänen et al., 2011; Tamminen et al., 2011a; Zhu et al., 2013; Muziasari et al., 2014). Of the 364 primer sets; 307 primer sets were used for ARGs encoding resistance to the nine main classes of antibiotics (aminoglycoside, beta-lactam, (flor)/(chlor)/(am)phenicol, macrolide (MSLB), multidrug-efflux, sulfonamide, tetracycline, trimethoprim, and vancomycin) and covering the three major mechanisms of antibiotic resistance (antibiotic efflux-pumps, cellular protection and antibiotic deactivation); 21 primer sets for other genes encoding resistance to antibacterial compounds, such as antiseptic and antibacterial peptides; 34 primer sets for genes associated with MGEs, such as plasmids, transposons, and insertion sequences (IS) as well as integrons; one primer set for a mercury resistance gene (*merA*) to study a possible co-selection of ARGs with mercury resistance gene; and one primer set for the 16S rRNA genes. Each primer set was designed to target sequence diversity within a gene to assess the environmental resistome and was therefore analyzed independently.

The qPCR array was performed in the SmartChip Real-time PCR system (WaferGen Biosystem, Freemont, CA, USA). In short, the SmartChip has 5184 reaction wells with a volume of 100 nL each, filled using the SmartChip Multisample Nanodispenser (WaferGen Biosystem, Freemont, CA, USA). PCR cycling conditions and initial data processing were as previously described (Wang et al., 2014). However, the detection limit was at a threshold cycle (C_T_) of 27 (Zhu et al., 2013; Karkman et al., 2016; Muziasari et al., 2016). The melting curve of each PCR product was also analyzed to monitor the specificity of the primer sets. The abundance of the detected genes in proportion to the 16S rRNA gene in each DNA sample was calculated using relative quantification with the 2^−ΔCT^ method, in which ΔC_T_ = (C_T_ detected gene − C_T_ 16S rRNA gene) (Schmittgen and Livak, 2008).

### Data analysis

To see if the MGEs are involved in the prevalence of ARGs, the correlation between the total relative abundances of transposases and the most prevalent ARG in the intestinal contents of the farmed fish were analyzed using linear regression. A *p* < 0.05 were considered to be significant. In addition, the notched box-plot was used to see the difference between the detected genes' relative abundances. The detected genes were grouped based on the three mechanisms of ARGs (antibiotic deactivation, cellular protection, and efflux-pumps), resistance genes to antiseptics, and MGE-associated genes. The notch in the box-plot displays the 95% confidence interval of the median (McGill et al., 1978). The linear regression and notched box-plot was plotted using a graphing package for R, ggplot2 v.0.9.3.1.

The 20 farmed-fish were grouped based on fish species (rainbow trout and whitefish), size, and age (small and big fish), farming time (3 and 15 months), and the history of antibiotic treatment at the farms (treated and non-treated) as shown in Table 1. To compare the resistome composition of the intestinal contents between the farmed fish groups, the genes' presence in each sample and their relative abundance values were used to calculate the distance matrix of non-metric multidimensional scaling (NMDS) or matrix of dissimilarities. The NMDS distance matrix between samples was performed based on the Bray-Curtis method using function metaMDS from the Vegan package in R (Oksanen et al., 2014). Values missing from the data due to no detection in the qPCR array were replaced with zero for the data analysis. Function ordiplot and ordiellipse from Vegan were used, respectively, to plot the NMDS distance matrix results and to add the 95% confidence region of the farmed fish groups. All analyses were performed using RStudio v.0.98.501 (RStudio, Boston, MA, 2012).

## Results

### Antibiotic resistance genes in the intestinal contents

**Figure 1:**
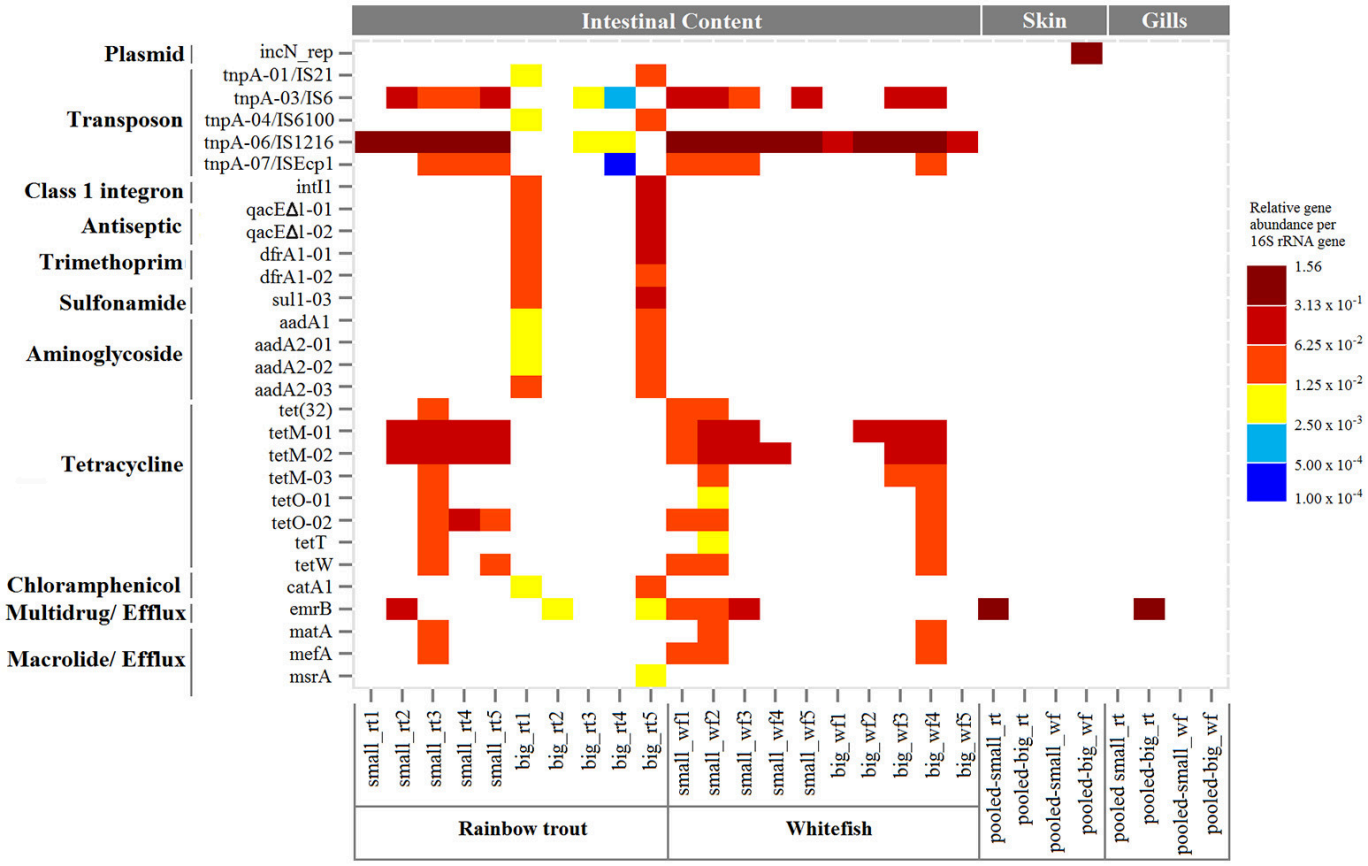
**Resistome composition of fish raised in the Northern Baltic Sea farms, Finland**. The Y axis presents the gene assays of the qPCR array grouped by mobile genetic element (MGE) and by classification of the antibiotics the genes confer resistance to. The X axis presents the farmed fish organized by the type of samples: the intestinal contents, mucosal skin, and gill filaments of small rainbow trout (small_rt), big rainbow trout (big_rt), small whitefish (small_wf), and big whitefish (big_wf). The color scale indicates five-fold changes in the genes' relative abundance in proportion to the 16S rRNA gene. White indicates that the respective gene was not detected or below the detection limit of each assay (C_T_ cut-off was at cycle 27) in the qPCR array.

We analyzed the ARG composition in the intestinal contents of 20 fish from two Northern Baltic Sea farms. The 20 farmed-fish consisted of five fish from each group of small rainbow trout, big rainbow trout, small whitefish, and big whitefish. The WaferGen qPCR array used 307 primer sets to detect and quantify ARGs, 21 primer sets for antibacterial or antiseptic resistance genes, 34 primer sets for MGE-associated genes, and one primer set for a mercury resistance gene. Altogether 28 out of the 363 genes were detected in the intestinal contents of the farmed-fish (Figure 1). The detected genes included transposases (*tnpA*) associated with IS21, IS6, IS6100, IS1216, and ISEcp1, an integrase gene of class 1 integron (*intI1*), a gene encoding resistance to an antiseptic which is also known as a backbone gene of class 1 integrons (*qacE*Δ*1*), a sulfonamide resistance gene (*sul1*), a trimethoprim resistance gene (*dfrA1*), tetracycline resistance genes [*tet(32), tetM, tetO, tetT*, and *tetW*], aminoglycoside resistance genes (*aadA1* and *aadA2*), a chloramphenicol resistance gene (*catA1*), a multidrug-efflux resistance gene (*emrB*), and macrolide (MLSB)-efflux resistance genes (*matA, mefA*, and *msrA*).

**Figure 2:**
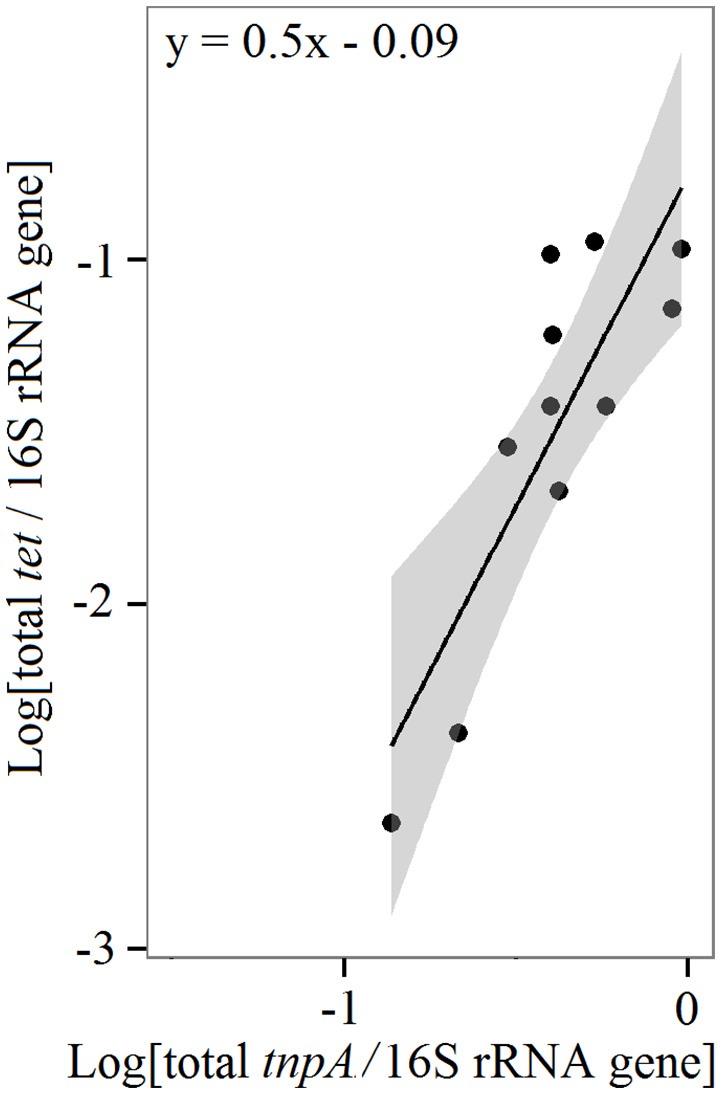
**Correlation analysis**. Linear regression model with log transformed variables between the transposases (*tnpA* genes) and tetracycline resistance genes (*tet* genes) in the intestinal contents of the fish farmed at the Northern Baltic Sea farms [*F*_(1, 9)_ = 20.3; *p* = 0.001; *R*^2^ = 0.71]. Each point presents the total relative abundances of the genes in proportion to the 16S rRNA gene in every intestinal content sample. The line indicates the regression model and the gray area the 95% confidence intervals.

Three of the 28 detected genes were found in all four groups of the farmed-fish: small rainbow trout (small_rt), big rainbow trout (big_rt), small whitefish (small_wf), and big whitefish (big_wf) (Figure S1). The three genes were all transposases that associated with IS1216, IS6, and ISEcp1. The transposase associated with IS1216 was the most abundant gene detected in almost every intestinal content with a relative abundance of ca. 10^−3^–10^−1^ in proportion to the 16S rRNA gene (Figure 1). The tetM-01 and tetM-02, targeting variants of the tetracycline resistance gene, *tetM*, showed these genes to be the most prevalent and abundant among the detected genes with relative abundances of ca. 10^−2^–10^−1^ in proportion to the 16S rRNA gene. Other tetracycline resistance genes [*tet(32), tetO, tetT*, and *tetW*] were found in at least one of the five intestinal contents from small_rt, small_wf, and big_wf. The total relative abundances of the *tet* genes and transposases were significantly correlated [*F*_(1, 9)_ = 22.3, *p* = 0.001, *R*^2^ = 0.71] indicating that the transposases could be connected to the prevalence of the *tet* genes in the intestinal contents (Figure 2). *tet* genes were not found in the big_rt samples. Two samples of the big rainbow trout, big_rt1 and big_rt5, differed from the rest of the intestinal content samples (Figures S1, S2) and carried genes that were not detected in the other intestinal content samples (Figure 1). These genes were transposases associated with IS21 and IS6100, *intI1, qacE*Δ*1, sul1, dfrA1, aadA1, aadA2*, and *catA1*. The efflux-pump resistance genes, *emrB, matA, mefA*, and *msrA*, were found in at least one of the intestinal contents. The mercury resistance gene (*merA*) was not detected in any of the samples.

**Figure 3:**
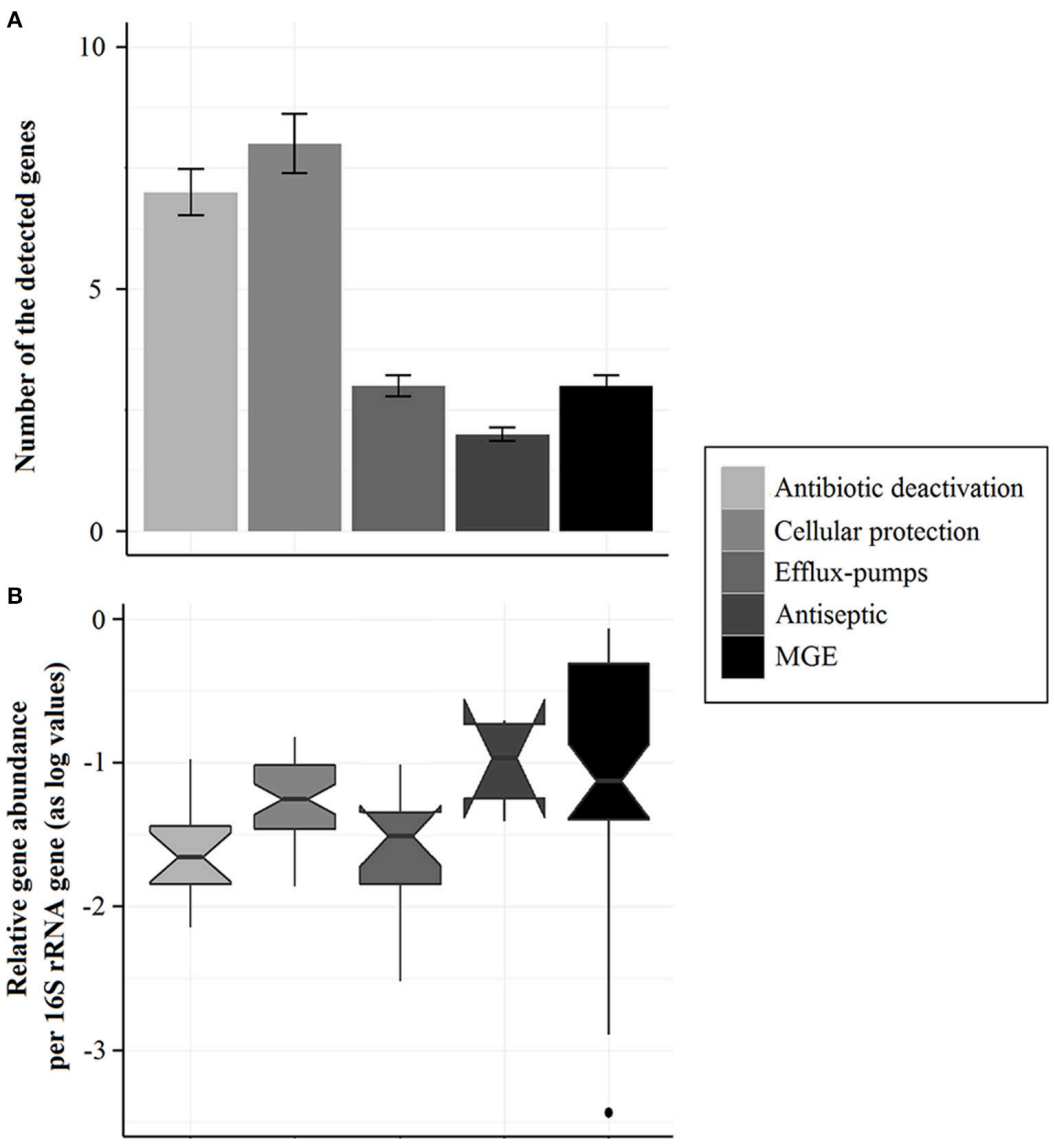
**(A)** Number of the detected genes. Bars represent the numbers of genes detected in the intestinal contents of the Baltic Sea farmed fish. Error bars indicate standard error (*n* = 20). The detected genes included ARGs, antiseptic resistance genes, and genes associated with mobile genetic elements (MGEs). The ARGs are grouped by the mechanism of resistance: antibiotic deactivation, cellular protection, and efflux-pumps. **(B)** Relative abundances of the genes detected in the farmed fish in proportion to the 16S rRNA gene (as log values). The box-plot presents the biological replicates of the farmed fish (*n* = 20) and the notch the 95% confidence intervals of the median.

In all, the detected ARGs in the intestinal contents of the farmed fish included the three major resistance mechanisms: antibiotic deactivation, cellular protection, and efflux pumps (Table S4, Figure 3A). The detected genes' relative abundances were ca. 10^−4^–10^−1^ in proportion to the 16S rRNA gene (Figure 3B, Tables S2, S3). Relative abundances of the MGE, cellular protection and antiseptic resistance genes were similar and higher compared to the efflux-pump and antibiotic deactivation resistance genes (Figure 3B).

We compared the resistome composition of the intestinal contents between groups of the farmed-fish (Table 1). The groups based on fish species, size, farming time, and the history of antibiotic treatment at the farms did not separate in the non-metric Bray-Curtis dissimilarity (Figures S2A–D). However, the intestinal content resistome of each individual fish within the groups seemed to have its own composition, as shown in Figure 1.

### Antibiotic resistance genes in the mucosal skin and gill filaments

The 364 assays of the WaferGen qPCR array were used to observe and quantify the resistome not only in the intestinal contents of the farmed fish but also in the fish mucosal skin and gill filaments. The skin and gill samples were pooled according to fish species and size to obtain a sufficient amount of DNA for the qPCR array analysis. In total, two genes were detected in the mucosal skin and only one gene in the gill filaments (Figure 1). In the mucosal skin, the two detected genes were *rep*, a specific replicon region of broad-host-range IncN plasmid, and *emrB*, a multidrug-efflux resistance gene. In the mucosal gill filaments, the *emrB* was the only detected gene. The *emrB* was the only gene found in each sample type: the intestinal contents, mucosal skin and gill filaments of the farmed fish.

## Discussion

### Farmed fish feces as a source of gene enrichment in sediments

There is growing concern that antibiotic use in fish farming promotes the enrichment of ARGs in the environment (Cabello et al., 2013). The prevalence of resistant bacteria carrying ARGs (Nonaka et al., 2007; Shah et al., 2014) and enrichment of ARGs in sediments below fish farms have been observed globally (Tamminen et al., 2011a; Gao et al., 2012; Muziasari et al., 2014, 2016; Xiong et al., 2015). However, the causes of ARG enrichment in the farm sediments have received very little attention. We assumed that farmed fish feces are a plausible source of ARG enrichment in sediments below fish farms in the Northern Baltic Sea, Finland. In this study, we analyzed the resistome composition of the intestinal contents of 20 fish raised at the Baltic Sea farms. We used the WaferGen qPCR array with 364 primer sets of known target genes. We compared the composition of the genes detected in the intestinal contents to our previous data of the sediment resistome at the farms (Tamminen et al., 2011a; Muziasari et al., 2014, 2016). We found that most of the detected genes in the intestinal contents (20 of the 28 genes) reflected the composition of the genes enriched in the farm sediments. Table 2 shows the 20 same genes that were detected in both farmed fish intestinal contents and farm sediments including transposases, class 1 integron-associated genes, and ARGs encoding resistance to tetracycline, sulfonamide, trimethoprim, and aminoglycoside. Average relative abundance of the genes in the intestinal contents was ca. 10^−2^–10^−1^ in proportion to the 16S rRNA gene and ca. 10^−4^–10^−3^ in proportion to the 16S rRNA gene in the farm sediments (Table 2). Bacterial community changes in the sediments in response to fish farming in the Baltic Sea also have been reported, with *Actinobacteria, Chloroflexi*, and *Firmicutes* prominent in the farm sediments (Tamminen et al., 2011b). *Actinobacteria and Firmicutes* are known as the core intestinal microbiota of salmonids (Pond et al., 2006; Navarrete et al., 2010; Wong et al., 2013). These findings provide indirect evidence supporting our assumption that certain ARGs are being introduced into the sediments below the fish farms in the Northern Baltic Sea via the bacteria contained in the feces of the farmed fish.

**Table 2:** **Twenty of the 28 genes detected in the farmed fish intestinal contents**.

**Classification of the antibiotics the genes confer resistance to**	**qPCR assay**	**Average relative abundance to the 16S rRNA gene**	
		**Fish intestinal contents (sampled in 2014)**	**Fish farm sediments sediments (sampled in 2006–2012)**
Aminoglycoside	aadA1	2 × 10^2^	^a^3 × 10^4^
Aminoglycoside	aadA2-01	2 × 10^2^	^a^2 × 10^4^
Aminoglycoside	aadA2-02	2 × 10^2^	^a^4 × 10^4^
Aminoglycoside	aadA2-03	4 × 10^2^	^a^8 × 10^4^
Trimethoprim	dfrA1	6 × 10^2^	^a^3 × 10^3^
Trimethoprim	dfrA1-02	4 × 10^2^	^b^1 × 10^3^
Class 1 integron	intI1	6 × 10^2^	^b^3 × 10^3^
Other (Antiseptic)	qacEΔ1-01	1 × 10^2^	^a^5 × 10^3^
Other (Antiseptic)	qacEΔ1-02	1 × 10^1^	^a^3 × 10^3^
Sulfonamide	sul1	7 × 10^2^	^b^4 × 10^3^
Tetracycline	tet(32)	3 × 10^2^	^a^1 × 10^3^
Tetracycline	tetM-01	1 × 10^1^	^a^3 × 10^3^
Tetracycline	tetM-02	9 × 10^2^	^a^2 × 10^3^
Tetracycline	tetM-03	4 × 10^2^	^c^1 × 10^3^
Tetracycline	tetO-01	2 × 10^2^	^a^2 × 10^3^
Tetracycline	tetW-01	4 × 10^2^	^a^4 × 10^4^
Transposon	tnpA-01	3 × 10^2^	^a^6 × 10^4^
Transposon	tnpA-04	4 × 10^2^	^a^2 × 10^4^
Transposon	tnpA-06	5 × 10^1^	^a^1 × 10^3^
Transposon	tnpA-07	4 × 10^2^	^a^4 × 10^3^

a *Muziasari et al. (2016)*.

b *Muziasari et al. (2014)*.

c *Tamminen et al. (2011a)*.

*^a, c^The quantification of the genes using standard qPCR*.

*The 20 genes were the same genes found to be enriched in the sediments below fish farms in the Northern Baltic Sea, Finland. The table also shows the average relative abundance of the genes to the 16S rRNA gene in the intestinal contents and in the farm sediments. The gene assays of the qPCR array grouped by classification of the antibiotics the genes confer resistance to, class 1 integron and transposon*.

The enrichment of ARGs also could be caused by the presence of antibiotics in the sediments. Although concentrations of tetracycline, oxytetracycline, sulfamethoxazole, sulfadiazine, and trimethoprim in the sediments were very low (ca. 1–100 ng/g of sediment), selection of resistance by antibiotics might have occurred in the past with the ARGs persisting in the sediments (Tamminen et al., 2011a; Muziasari et al., 2014). Enrichment of ARGs in the absence of selective antibiotic pressure could also be caused by co-selection with heavy metals (Baker-Austin et al., 2006). While mercury can be found in fish feed (Choi and Cech, 1998), biological and chemical analyses of the sediments revealed only very low background concentrations of this metal (Pitkänen et al., 2011). However, other heavy metals (copper, cadmium, zinc) can potentially be present in fish feed or antifouling substances and exert co-selective pressures in these sediments (Seiler and Berendonk, 2012).

On the other hand, the mercury resistance gene *merA* was not detected in the intestinal contents, but it was enriched in the farm sediments (Pitkänen et al., 2011). Similarly, not all sediment-enriched ARGs were detected in the intestinal contents. This might be due to the amount of the genes in the intestinal contents that were below the detection limit of the qPCR array, which varies between the primer sets (ca. 10^−2^–10^−4^ copy number of target genes). Alternatively, the enriched genes in the farm sediments might be coming from other sources such as uneaten medicated feed (Kerry et al., 1995). The deposition of uneaten feed that increases the amount of organic matter in the farm sediments might be also resulting in the enrichment of indigenous sediment bacteria carrying the enriched ARGs (Tamminen et al., 2011b; Buschmann et al., 2012).

Because the ARGs were detected in the intestinal contents of the farmed fish with no history of antibiotic treatment at the farms, the ARGs could potentially be derived from resistant bacteria in the surrounding water environment that were able to be transferred to the intestinal microbiota of fish (Giatsis et al., 2015). The ARGs also may have already been selected in the fish intestine before entering the Baltic Sea farm cages, possibly during the processes of hatching or rearing juvenile fish in freshwater ponds. Specific record of antibiotics use in the juvenile fish prior to entering the Baltic Sea farms was not available. However, it has been reported that antibiotics were used against *F. psychrophilum* and *F. columnare* in Finnish hatchery farms (EVIRA, 2007). Antibiotic resistant bacteria can be found in fish eggs and juvenile fish due to the routine use of antibiotics during the rearing process (Hansen and Olafsen, 1999). The ARGs are still present in the fish intestine even after transferred to different water environments at the Baltic Sea farms, maybe because the resistant bacteria carrying the ARGs are the core intestinal microbiota of the farmed fish (Ghanbari et al., 2015). Although our data does not refute the prior understanding on the occurrence of antibiotic resistance in the fish farming environment due to antibiotic use at farms, it suggests that the juvenile fish may have a greater role as the source of antibiotic resistance than has been expected before. The routine use of antibiotics in fish hatchery and rearing farms should not be recommended because it may affect the natural microbiota of fish larvae and juvenile (Ringø et al., 1995). It is, therefore, important to monitor the antibiotic treatments of juvenile fish to minimize the potential risk of ARG emergence in fish farm environments.

### Resistome composition of the intestinal contents

Tetracycline resistance (*tet*) genes were the most prevalent ARGs in the intestinal contents of the farmed fish. The detected *tet* genes were genes which only encode ribosomal protection proteins (RPP), the *tet(32), tetM, tetO, tetT*, and *tetW*. *tetM* was found to be the most prevalent of the detected ARGs. This was expected, since *tetM* has the widest host range of all known *tet* genes and is well known to be associated with a very wide range of conjugative transposons (Roberts, 2005). Beside *tetM* our results were in contrast to other studies in which the efflux-pump genes, *tetA, tetD*, and *tetE* were commonly found in resistant bacteria isolated from intestines of farmed fish (Furushita et al., 2003; Akinbowale et al., 2007). The transposase-IS1216 is predominant in the intestinal contents of the Baltic Sea farmed fish and known to be associated with conjugative transposons (Ciric et al., 2011). The RPP genes are usually associated with conjugative transposons, while the efflux-pump genes are generally associated with large plasmids (Roberts, 2012). Moreover, we observed a significant correlation between the total relative abundances of the *tet* genes and transposases (*p* = 0.001, *R*^2^ = 0.71). Transposases are, therefore, likely involved in the prevalence of the *tet* genes in the Baltic Sea farmed fish intestinal contents. A highly significant correlation between the abundance of transposases and *tet* genes has been observed also in swine manure (Zhu et al., 2013).

In this study, the *tet* genes were not found in the intestinal contents of the big rainbow trout. However, some genes were only found in the intestinal contents of two big rainbow trout. Those genes were the transposases associated with IS21 or Tn21 and IS6100, genes associated with class 1 integrons, *intI1* and *qacE*Δ*1*, and the ARGs *sul1, dfrA1, aadA1, aadA2*, and *catA1*. Class 1 integrons are known to be able to capture and incorporate gene cassettes consisting of *sul1, dfrA1, aadA1, aadA2* (Partridge et al., 2009), and *catA1* (Soto et al., 2003). In the farm sediments, class 1 integrons carry the *sul1* and *aadA1* genes (Pärnänen et al., 2016). This explains the presence of *aadA1* aminoglycoside resistance genes at the farms even though aminoglycosides have never been used there. The prevalence of class 1 integrons could be cause by the association of class 1 integrons with Tn21 (Liebert et al., 1999) or with IS6100 (Partridge et al., 2001). The presence of *sul1, dfrA1, aadA1, aadA2*, and *catA1* in the intestinal contents of two big rainbow trout may have been mediated by the class 1 integrons carried by MGEs such as Tn21 or IS6100.

The chloramphenicol and macrolide resistance genes were found in the intestinal contents and are also known to be associated with conjugative transposons (Hegstad et al., 2010). This suggests that the presence of ARGs in the farmed fish intestinal contents may be connected to the prevalence of transposases. Otherwise, the ARGs, especially efflux-pump resistance genes, could be intrinsically possessed by fish bacteria since the efflux-pump resistance genes are also known to have other functions beside antibiotic resistance, such as intracellular metabolite detoxification (Martinez et al., 2009).

We observed that the resistome composition of the intestinal contents varied between individual farmed fish. Beside the connection with the MGEs, the resistome composition in the fish intestine may be also correlated with the composition of bacterial communities (Forsberg et al., 2014). It has been reported that the bacterial communities in the intestines of individual farmed fish varies greatly and stable regardless of the variations of diet and rearing system (Wong et al., 2013; Lyons et al., 2017). Our results also show that the resistome composition of the intestinal contents might not connected to the types of fish species, size, farming time, and antibiotic treatments at the farms. This suggests that the resistome of the farmed-fish intestinal contents has its own composition and is influenced less by the fish farming processes. However, these results may vary if more farmed fish are sampled and included in the future studies. To our knowledge, this is the first study reporting the resistome composition of farmed-fish using a culture-independent method.

The intestinal content resistome of the farmed-fish covered the three mechanisms of resistance genes: antibiotic deactivation, cellular protection, and efflux-pumps. The relative abundance of cellular protection resistance genes was higher than the other two mechanisms of resistance genes. This is because the cellular protection resistance genes included all the detected RPP-tetracycline resistance genes that were the most abundant ARGs in the intestinal contents of the farmed-fish. In the farm sediment resistome, however, the efflux-pump resistance genes were the most abundant (Muziasari et al., 2016). The low frequency of detection of efflux-pump resistance genes in the resistomes of farmed-fish intestines in this study might be due to the qPCR array targeting only known ARGs since the qPCR array requires prior knowledge of ARG sequences for primer design. Insight on the ARG mechanisms within fish farming-associated resistomes is essential for predicting the emergence of ARGs at the fish farm facilities (Miranda et al., 2013).

### Resistome composition in the mucosal skin and gill filaments

In the mucosal skin and gill filaments of the farmed-fish, only one of the 307 targeted ARGs, the multidrug-efflux resistance gene, *emrB*, was detected. The *emrB* was also detected in the intestinal contents. Since the *emrB* was found in the three different sample types taken from the farmed fish, the *emrB* gene maybe intrinsically possessed by fish bacteria (Martinez et al., 2009). In other studies, ARGs encoding resistance to tetracycline, sulfonamide, and trimethoprim have been frequently found in resistant bacteria isolated from the skin and gills of farmed fish (Schmidt et al., 2001; Akinbowale et al., 2007). The different results might be due to the difference between ARG detection methods, as the qPCR array detection limit may prevent detection of a low abundance of ARGs in fish mucosal skin and gill filaments.

## Conclusion

This study indicates that the resistomes of the intestinal contents of farmed fish contribute to the enrichment of ARGs in sediments below fish farms in the Northern Baltic Sea. Using high-throughput methods to study fish resistomes, we found a significant correlation between the relative abundances of the transposases in them and tetracycline resistance genes. This might lead to the risk of ARG mobilization from piscine bacteria to bacteria in the surrounding environments. The presence of transposases and class 1 integrons might also affect the prevalence of certain ARGs in the intestinal contents of individual fish and shape the composition of their resistomes. Determining the sources of ARGs at fish farms is thus important for managing and minimizing the emergence of ARGs at the fish farms and for lowering the risk of ARG spreading to the surrounding farm environments.

## Author contributions

WM and MV: contributions to the experimental design. WM and LP: sampling. WM, LP, and RS: acquisition and analysis of data for the work. HS, JT, and MV: analysis, interpretation and adding important intellectual content. WM, LP, HS, RS, JT, and MV: drafting and revising the manuscript.

## Funding

Academy of Finland grants, Maj and Tor Nessling Foundation and with support from the Center for Microbial Ecology and the Center for Health Impacts of Agriculture at Michigan State University.

### Conflict of interest statement

The authors declare that the research was conducted in the absence of any commercial or financial relationships that could be construed as a potential conflict of interest.

## Supplementary material

Figure S1 **Venn diagram**. Number of genes shared by the groups of the Baltic Sea farmed fish: small rainbow trout (small_rt), big rainbow trout (big_rt), small whitefish (small_whitefish), and big whitefish (big_wf). Each group consisted of five farmed fish.

Figure S2 **Non-metric multidimensional scaling (NMDS) of the intestinal content resistomes of fish farmed at the Baltic Sea farms**. The distance matrix between the intestinal content resistomes was based on Bray-Curtis dissimilarity. The farmed fish (*n* = 20) were grouped based on **(A)** Fish species: rainbow trout (dark red) and whitefish (rose). **(B)** Fish size: big fish (dark red) and small fish (rose). **(C)** Farming time at the farms: 3 months (dark red) and 15 months (rose). **(D)** The history of antibiotic treatment at the farms: Non-treated (dark red) and treated with a combination of sulfonamide-trimethoprim 1 month before the fish were sampled (rose). Ellipse line indicates 95% confidence regions of the farmed fish groups.

Table S1 **The list of primer sets used in the qPCR array**.

Table S2 **Gene abundance values relative to the 16S rRNA gene in Rainbow trout raised at the Northern Baltic Sea farms**.

Table S3 **Gene abundance values relative to the 16S rRNA gene in European whitefish raised at the Northern Baltic Sea farms**.

Table S4 **Number of positive qPCR assays and the total assays**.
