# Corrigendum: The Resistome of Farmed Fish Feces Contributes to the Enrichment of Antibiotic Resistance Genes in Sediments below Baltic Sea Fish Farms

**DOI:** 10.3389/fmicb.2017.01491

**Published:** 2017-08-02

**Authors:** Windi I. Muziasari, Leena K. Pitkänen, Henning Sørum, Robert D. Stedtfeld, James M. Tiedje, Marko Virta

**Affiliations:** ^1^Department of Food and Environmental Sciences, University of Helsinki Helsinki, Finland; ^2^Department of Food Safety and Infection Biology, Norwegian University of Life Sciences Oslo, Norway; ^3^Department of Civil and Environmental Engineering, Center for Microbial Ecology, Michigan State University Michigan, MI, USA

**Keywords:** rainbow trout, whitefish, class 1 integrons, transposons, mobile genetic elements, qPCR array, culture-independent method

In the original article, there was a mistake in Table 2. Twenty of the 28 genes detected in the farmed fish intestinal contents as published. In the Table 2, the numbers of average relative abundance of the 20 genes to the 16S rRNA gene were not correct. The corrected numbers of the gene abundances appears below. We apologize for this error and the error does not change the scientific conclusions of the article in any way.

**Table 2:** Twenty of the 28 genes detected in the farmed fish intestinal contents.

**Classification of the antibiotics the genes confer resistance to**	**qPCR assay**	**Average relative abundance to the 16S rRNA gene**	
		**Fish intestinal contents (sampled in 2014)**	**Fish farm sediments (sampled in 2006–2012)**
Aminoglycoside	aadA1	2 × 10^−2^	^a^3 × 10^−4^
Aminoglycoside	aadA2-01	2 × 10^−2^	^a^2 × 10^−4^
Aminoglycoside	aadA2-02	2 × 10^−2^	^a^4 × 10^−4^
Aminoglycoside	aadA2-03	4 × 10^−2^	^a^8 × 10^−4^
Trimethoprim	dfrA1	6 × 10^−2^	^a^3 × 10^−3^
Trimethoprim	dfrA1-02	4 × 10^−2^	^b^1 × 10^−3^
Class 1 integron	intI1	6 × 10^−2^	^b^3 × 10^−3^
Other (Antiseptic)	qacEΔ1-01	1 × 10^−2^	^a^5 × 10^−3^
Other (Antiseptic)	qacEΔ1-02	1 × 10^−1^	^a^3 × 10^−3^
Sulfonamide	sul1	7 × 10^−2^	^b^4 × 10^−3^
Tetracycline	tet(32)	3 × 10^−2^	^a^1 × 10^−3^
Tetracycline	tetM-01	1 × 10^−1^	^a^3 × 10^−3^
Tetracycline	tetM-02	9 × 10^−2^	^a^2 × 10^−3^
Tetracycline	tetM-03	4 × 10^−2^	^c^1 × 10^−3^
Tetracycline	tetO-01	2 × 10^−2^	^a^2 × 10^−3^
Tetracycline	tetW-01	4 × 10^−2^	^a^4 × 10^−4^
Transposon	tnpA-01	3 × 10^−2^	^a^6 × 10^−4^
Transposon	tnpA-04	4 × 10^−2^	^a^2 × 10^−4^
Transposon	tnpA-06	5 × 10^−1^	^a^1 × 10^−3^
Transposon	tnpA-07	4 × 10^−2^	^a^4 × 10^−3^

a *Muziasari et al., 2016*.

b *Muziasari et al., 2014*.

c *Tamminen et al., 2011*.

*^b,c^The quantification of the genes used standard qPCR*.

*The 20 genes were the same genes found to be enriched in the sediments below fish farms in the Northern Baltic Sea, Finland. The table also shows the average relative abundance of the genes to the 16S rRNA gene in the intestinal contents and in the farm sediments. The gene assays of the qPCR array grouped by classification of the antibiotics the genes confer resistance to, class 1 integron and transposon*.

## Conflict of interest statement

The authors declare that the research was conducted in the absence of any commercial or financial relationships that could be construed as a potential conflict of interest.

