# Supplementary figures and images for: The Resistome of Farmed Fish Feces Contributes to the Enrichment of Antibiotic Resistance Genes in Sediments below Baltic Sea Fish Farms
Source: Front Microbiol. 2017 Jan 6;7:2137. doi: 10.3389/fmicb.2016.02137 (PMC5216021; doi:10.3389/fmicb.2016.02137)

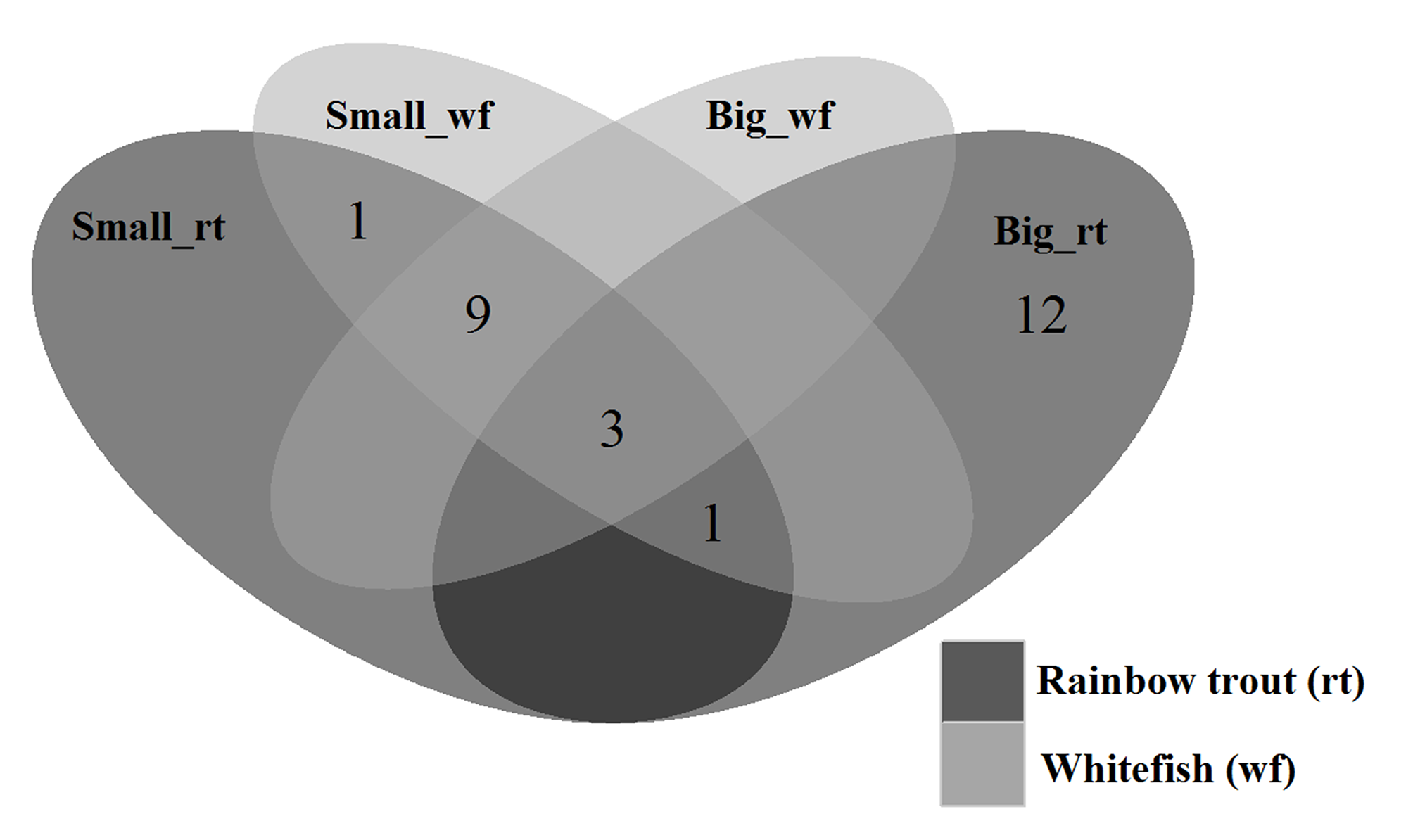

Supplement: Figure S1 — Venn diagram. Number of genes shared by the groups of the Baltic Sea farmed fish: small rainbow trout (small_rt), big rainbow trout (big_rt), small whitefish (small_whitefish), and big whitefish (big_wf). Each group consisted of five farmed fish. [file Image1.TIF]

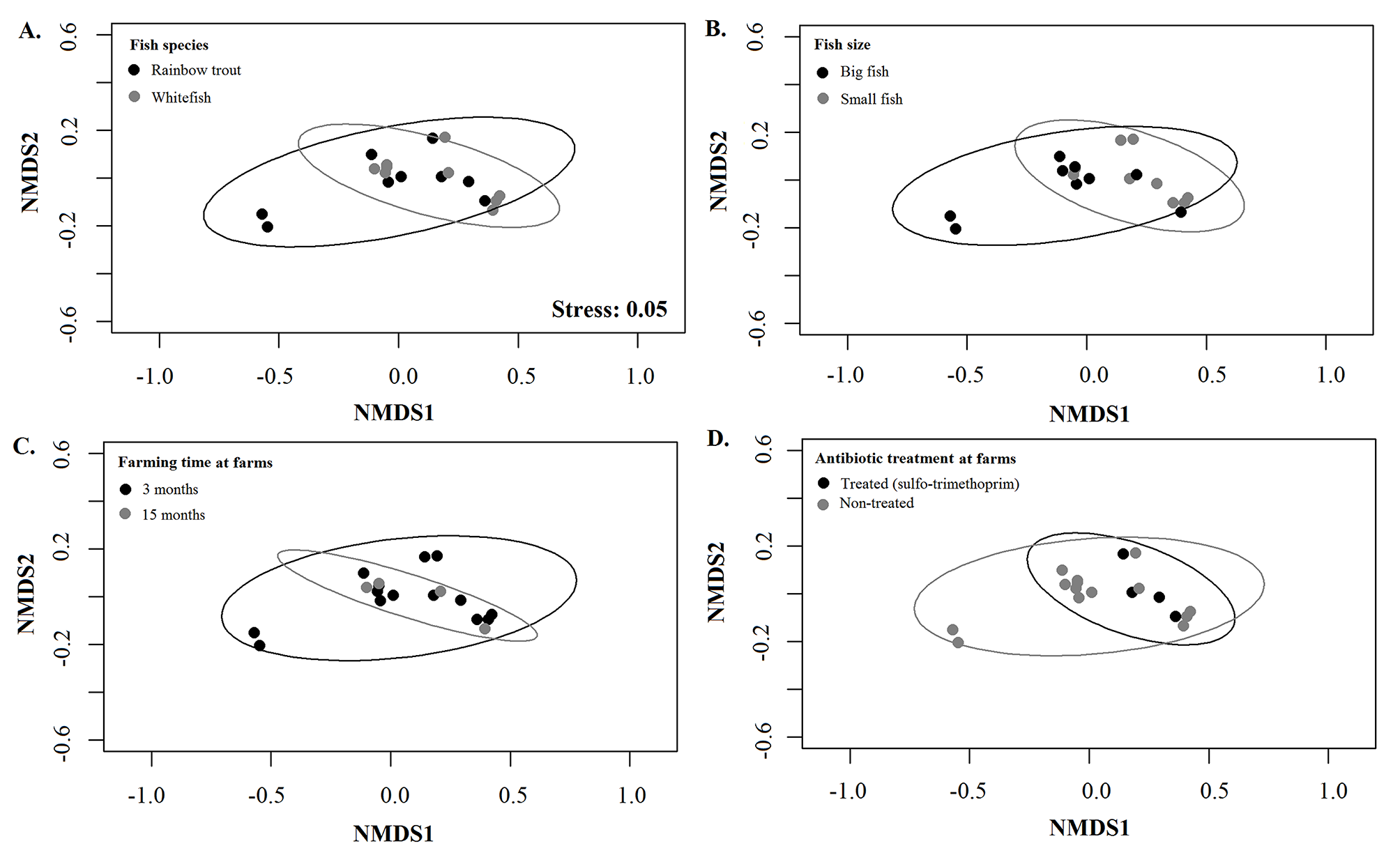

Supplement: Figure S2 — Non-metric multidimensional scaling (NMDS) of the intestinal content resistomes of fish farmed at the Baltic Sea farms. The distance matrix between the intestinal content resistomes was based on Bray-Curtis dissimilarity. The farmed fish (n = 20) were grouped based on (A) Fish species: rainbow trout (dark red) and whitefish (rose). (B) Fish size: big fish (dark red) and small fish (rose). (C) Farming time at the farms: 3 months (dark red) and 15 months (rose). (D) The history of antibiotic treatment at the farms: Non-treated (dark red) and treated with a combination of sulfonamide-trimethoprim 1 month before the fish were sampled (rose). Ellipse line indicates 95% confidence regions of the farmed fish groups. [file Image2.TIF]
