# Supplementary material for: The Resistome of Farmed Fish Feces Contributes to the Enrichment of Antibiotic Resistance Genes in Sediments below Baltic Sea Fish Farms
Source: Front Microbiol. 2017 Jan 6;7:2137. doi: 10.3389/fmicb.2016.02137 (PMC5216021; doi:10.3389/fmicb.2016.02137)
Supplement: Table S1 — The list of primer sets used in the qPCR array. [file DataSheet1.DOCX]

Supplementary Material

**Farmed fish feces as a plausible source of antibiotic resistance gene enrichment in Baltic Sea fish farm sediments**

Windi I. Muziasari, Leena K. Pitkänen, Henning Sørum, Robert D. Stedtfeld, James M. Tiedje, Marko Virta*

*** Correspondence:** Corresponding Authors: [marko.virta@helsinki.fi](mailto:marko.virta@helsinki.fi); [windi.muziasari@helsinki.fi](mailto:windi.muziasari@helsinki.fi)

# Supplementary Tables

| Table S1. The list of primer sets used in the qPCR array |
| --- |

| Assay Number | Assay Name | Forward Primer | Reverse Primer | Target gene | Classification of the antibiotics the gene confers resistance to | Mechanism of resistance |
| --- | --- | --- | --- | --- | --- | --- |
| 1 | 16S rRNA universal1 | GGGTTGCGCTCGTTGC | ATGGYTGTCGTCAGCTCGTG | *16S rRNA* | Other (Housekeeping) | other (housekeeping) |
| 2 | 16S rRNA universal2 | CCTACGGGAGGCAGCAG | ATTACCGCGGCTGCTGGC | *16S rRNA* | Other (Housekeeping) | other (housekeeping) |
| 3 | aac(6')-I | GACCGGATTAAGGCCGATG | CTTGCCTTGATATTCAGTTTTTATAACCA | *aac(6')-I* | Aminoglycoside | antibiotic deactivation |
| 4 | aac(6')-Ib-01 | GTTTGAGAGGCAAGGTACCGTAA | GAATGCCTGGCGTGTTTGA | *aac(6')-Ib* | Aminoglycoside | antibiotic deactivation |
| 5 | aac(6')-Ib-02 | CGTCGCCGAGCAACTTG | CGGTACCTTGCCTCTCAAACC | *aac(6')-Ib* | Aminoglycoside | antibiotic deactivation |
| 6 | aac(6')-Ib-03 | AGAAGCACGCCCGACACTT | GCTCTCCATTCAGCATTGCA | *aac(6')-Ib* | Aminoglycoside | antibiotic deactivation |
| 7 | aac(6')-Iy | GCTTTGCGGATGCCTCAAT | GGAGAACAAAAATACCTTCAAGGAAA | *aac(6')-Iy* | Aminoglycoside | antibiotic deactivation |
| 8 | aac(6')-II | CGACCCGACTCCGAACAA | GCACGAATCCTGCCTTCTCA | *aac(6')-II* | Aminoglycoside | antibiotic deactivation |
| 9 | aacA | AGAGCCTTGGGAAGATGAAGTTT | TTGATCCATACCATAGACTATCTCATCA | *aacA* | Aminoglycoside | antibiotic deactivation |
| 10 | aacC | CGTCACTTATTCGATGCCCTTAC | GTCGGGCGCGGCATA | *aacC* | Aminoglycoside | antibiotic deactivation |
| 11 | aacC2 | ACGGCATTCTCGATTGCTTT | CCGAGCTTCACGTAAGCATTT | *aacC2* | Aminoglycoside | antibiotic deactivation |
| 12 | aacC4 | CGGCGTGGGACACGAT | AGGGAACCTTTGCCATCAACT | *aacC4* | Aminoglycoside | antibiotic deactivation |
| 13 | aadA-01 | GTTGTGCACGACGACATCATT | GGCTCGAAGATACCTGCAAGAA | *aadA* | Aminoglycoside | antibiotic deactivation |
| 14 | aadA-02 | CGAGATTCTCCGCGCTGTA | GCTGCCATTCTCCAAATTGC | *aadA* | Aminoglycoside | antibiotic deactivation |
| 15 | aadA1 | AGCTAAGCGCGAACTGCAAT | TGGCTCGAAGATACCTGCAA | *aadA1* | Aminoglycoside | antibiotic deactivation |
| 16 | aadA2-01 | ACGGCTCCGCAGTGGAT | GGCCACAGTAACCAACAAATCA | *aadA2* | Aminoglycoside | antibiotic deactivation |
| 17 | aadA2-02 | CTTGTCGTGCATGACGACATC | TCGAAGATACCCGCAAGAATG | *aadA2* | Aminoglycoside | antibiotic deactivation |
| 18 | aadA2-03 | CAATGACATTCTTGCGGGTATC | GACCTACCAAGGCAACGCTATG | *aadA2* | Aminoglycoside | antibiotic deactivation |
| 19 | aadA5-01 | ATCACGATCTTGCGATTTTGCT | CTGCGGATGGGCCTAGAAG | *aadA5* | Aminoglycoside | antibiotic deactivation |
| 20 | aadA5-02 | GTTCTTGCTCTTGCTCGCATT | GATGCTCGGCAGGCAAAC | *aadA5* | Aminoglycoside | antibiotic deactivation |
| 21 | aadA9-01 | CGCGGCAAGCCTATCTTG | CAAATCAGCGACCGCAGACT | *aadA9* | Aminoglycoside | antibiotic deactivation |
| 22 | aadA9-02 | GGATGCACGCTTGGATGAA | CCTCTAGCGGCCGGAGTATT | *aadA9* | Aminoglycoside | antibiotic deactivation |
| 23 | aadD | CCGACAACATTTCTACCATCCTT | ACCGAAGCGCTCGTCGTATA | *aadD* | Aminoglycoside | antibiotic deactivation |
| 24 | aadE | TACCTTATTGCCCTTGGAAGAGTTA | GGAACTATGTCCCTTTTAATTCTACAATCT | *aadE* | Aminoglycoside | antibiotic deactivation |
| 25 | acc | CCCTGCGTTGTGGCTATGT | TTGGCCACGCCAATCC | *acc* | Aminoglycoside | antibiotic deactivation |
| 26 | acrA-01 | CAACGATCGGACGGGTTTC | TGGCGATGCCACCGTACT | *acrA* | Multidrug/ Efflux | efflux-pumps |
| 27 | acrA-02 | GGTCTATCACCCTACGCGCTATC | GCGCGCACGAACATACC | *acrA* | Multidrug/ Efflux | efflux-pumps |
| 28 | acrA-03 | CAGACCCGCATCGCATATT | CGACAATTTCGCGCTCATG | *acrA* | Multidrug/ Efflux | efflux-pumps |
| 29 | acrA-04 | TACTTTGCGCGCCATCTTC | CGTGCGCGAACGAACAT | *acrA* | Multidrug/ Efflux | efflux-pumps |
| 30 | acrA-05 | CGTGCGCGAACGAACA | ACTTTGCGCGCCATCTTC | *acrA* | Multidrug/ Efflux | efflux-pumps |
| 31 | acrB-01 | AGTCGGTGTTCGCCGTTAAC | CAAGGAAACGAACGCAATACC | *acrB* | Multidrug/ Efflux | efflux-pumps |
| 32 | acrB-02 | TGGTAGTGGGCGTCATTAACAC | GGCAACGTAATCCGAAATATCC | *acrB* | Multidrug/ Efflux | efflux-pumps |
| 33 | acrF | GCGGCCAGGCACAAAA | TACGCTCTTCCCACGGTTTC | *acrF* | Multidrug/ Efflux | efflux-pumps |
| 34 | acrR-01 | GCGCTGGAGACACGACAAC | GCCTTGCTGCGAGAACAAA | *acrR* | Multidrug/ Efflux | efflux-pumps |
| 35 | acrR-02 | GATGATACCCCCTGCTGTGAGA | ACCAAACAAGAAGCGCAAGAA | *acrR* | Multidrug/ Efflux | efflux-pumps |
| 36 | acrR-03 | TGCAACACGCGCTTTCTC | ACGATTGCGGGCAGGTT | *acrR* | Multidrug/ Efflux | efflux-pumps |
| 37 | adeA | CAGTTCGAGCGCCTATTTCTG | CGCCCTGACCGACCAAT | *adeA* | Multidrug/ Efflux | efflux-pumps |
| 38 | ampC-01 | TGGCGTATCGGGTCAATGT | CTCCACGGGCCAGTTGAG | *ampC* | Beta lactam | antibiotic deactivation |
| 39 | ampC-02 | GCAGCACGCCCCGTAA | TGTACCCATGATGCGCGTACT | *ampC* | Beta lactam | antibiotic deactivation |
| 40 | ampC-03 | AACAAAAGATCCCCGGTATGG | ACGCCCGTAAATGTTTTGCT | *ampC* | Beta lactam | antibiotic deactivation |
| 41 | ampC-04 | TCCGGTGACGCGACAGA | CAGCACGCCGGTGAAAGT | *ampC* | Beta lactam | antibiotic deactivation |
| 42 | ampC-05 | CTGTTCGAGCTGGGTTCTATAAGTAAA | CAGTATCTGGTCACCGGATCGT | *ampC* | Beta lactam | antibiotic deactivation |
| 43 | ampC-06 | CCGCTCAAGCTGGACCATAC | CCATATCCTGCACGTTGGTTT | *ampC* | Beta lactam | antibiotic deactivation |
| 44 | ampC-07 | CCGCCCAGAGCAAGGACTA | GCTCGACTTCACGCCGTAAG | *ampC* | Beta lactam | antibiotic deactivation |
| 45 | ampC-08 | GCAGCGAAGCGTCAGTCA | AGATCCGTGGCCGCATAA | *ampC* | Beta lactam | antibiotic deactivation |
| 46 | ampC-09 | CAGCCGCTGATGAAAAAATATG | CAGCGAGCCCACTTCGA | *ampC* | Beta lactam | antibiotic deactivation |
| 47 | aph | TTTCAGCAAGTGGATCATGTTAAAAT | CCAAGCTGTTTCCACTGTTTTTC | *aph* | Aminoglycoside | antibiotic deactivation |
| 48 | aph(2')-Id-01 | TGAGCAGTATCATAAGTTGAGTGAAAAG | GACAGAACAATCAATCTCTATGGAATG | *aph(2')-Id* | Aminoglycoside | antibiotic deactivation |
| 49 | aph(2')-Id-02 | TAAGGATATACCGACAGTTTTGGAAA | TTTAATCCCTCTTCATACCAATCCATA | *aph(2')-Id* | Aminoglycoside | antibiotic deactivation |
| 50 | aph6ia | CCCATCCCATGTGTAAGGAAA | GCCACCGCTTCTGCTGTAC | *aph6ia* | Aminoglycoside | antibiotic deactivation |
| 51 | aphA1 | TGAACAAGTCTGGAAAGAAATGCA | CCTATTAATTTCCCCTCGTCAAAAA | *aphA1* | Aminoglycoside | antibiotic deactivation |
| 52 | aphA3-01 | AAAAGCCCGAAGAGGAACTTG | CATCTTTCACAAAGATGTTGCTGTCT | *aphA3* | Aminoglycoside | antibiotic deactivation |
| 53 | aphA3-02 | CGGAATTGAAAAAACTGATCGAA | ATACCGGCTGTCCGTCATTT | *aphA3* | Aminoglycoside | antibiotic deactivation |
| 54 | bacA-01 | CGGCTTCGTGACCTCGTT | ACAATGCGATACCAGGCAAAT | *bacA* | Other^1^ | other |
| 55 | bacA-02 | TTCCACGACACGATTAAGTCATTG | CGGCTCTTTCGGCTTCAG | *bacA* | Other^1^ | other |
| 56 | bexA | GCGGATCTCTGGTCAGCAA | TGATTGATGGTTCCCCGTACA | *bexA* | Multidrug/ Efflux | efflux-pumps |
| 57 | bla1 | GCAAGTTGAAGCGAAAGAAAAGA | TACCAGTATCAATCGCATATACACCTAA | *bla1* | Beta lactam | antibiotic deactivation |
| 58 | blaACC | CACACAGCTGATGGCTTATCTAAAA | AATAAACGCGATGGGTTCCA | *blaACC* | Beta lactam | antibiotic deactivation |
| 59 | blaCMY | CCGCGGCGAAATTAAGC | GCCACTGTTTGCCTGTCAGTT | *blaCMY* | Beta lactam | antibiotic deactivation |
| 60 | blaCMY2-01 | AAAGCCTCAT GGGTGCATAAA | ATAGCTTTTGTTTGCCAGCATCA | *blaCMY2* | Beta lactam | antibiotic deactivation |
| 61 | blaCMY2-02 | GCGAGCAGCCTGAAGCA | CGGATGGGCTTGTCCTCTT | *blaCMY2* | Beta lactam | antibiotic deactivation |
| 62 | blaCTX-M-01 | GGAGGCGTGACGGCTTTT | TTCAGTGCGATCCAGACGAA | *blaCTX-M* | Beta lactam | antibiotic deactivation |
| 63 | blaCTX-M-02 | GCCGCGGTGCTGAAGA | ATCGGATTATAGTTAACCAGGTCAGATTT | *blaCTX-M* | Beta lactam | antibiotic deactivation |
| 64 | blaCTX-M-03 | CGATACCACCACGCCGTTA | GCATTGCCCAACGTCAGATT | *blaCTX-M* | Beta lactam | antibiotic deactivation |
| 65 | blaCTX-M-04 | CTTGGCGTTGCGCTGAT | CGTTCATCGGCACGGTAGA | *blaCTX-M* | Beta lactam | antibiotic deactivation |
| 66 | blaCTX-M-05 | GCGATAACGTGGCGATGAAT | GTCGAGACGGAACGTTTCGT | *blaCTX-M* | Beta lactam | antibiotic deactivation |
| 67 | blaCTX-M-06 | CACAGTTGGTGACGTGGCTTAA | CTCCGCTGCCGGTTTTATC | *blaCTX-M* | Beta lactam | antibiotic deactivation |
| 68 | blaCTX-M-07 | CGTCACGCTGTTGTTAGGAA | CGCTCATCAGCACGATAAAG | *blaCTX-M* | Beta lactam | antibiotic deactivation |
| 69 | blaCTX-M-08 | CGATGTGCAGTACCAGTAA | GCAATGGGATTGTAGTTAA | *blaCTX-M* | Beta lactam | antibiotic deactivation |
| 70 | blaDHA | TGGCCGCAGCAGAAAGA | CCGTTTTATGCACCCAGGAA | *blaDHA* | Beta lactam | antibiotic deactivation |
| 71 | blaGES | GCAATGTGCTCAACGTTCAAG | GTGCCTGAGTCAATTCTTTCAAAG | *blaGES* | Beta lactam | antibiotic deactivation |
| 72 | blaIMP-01 | AACACGGTTTGGTGGTTCTTGTA | GCGCTCCACAAACCAATTG | *blaIMP* | Beta lactam | antibiotic deactivation |
| 73 | blaIMP-02 | AAGGCAGCATTTCCTCTCATTTT | GGATAGATCGAGAATTAAGCCACTCT | *blaIMP* | Beta lactam | antibiotic deactivation |
| 74 | blaIMP-03 | GGAATAGAGTGGCTTAATTC | GGTTTAACAAAACAACCACC | *blaIMP* | Beta lactam | antibiotic deactivation |
| 75 | blaKPC-01 | TCGCCCTGGATGTACACCTT | ACCATTGCCGACATCAACAAC | *blaKPC* | Beta lactam | antibiotic deactivation |
| 76 | blaKPC-02 | CAGCTCATTCAAGGGCTTTC | GGCGGCGTTATCACTGTATT | *blaKPC* | Beta lactam | antibiotic deactivation |
| 77 | blaKPC-03 | GCCGCCGTGCAATACAGT | GCCGCCCAACTCCTTCA | *blaKPC* | Beta lactam | antibiotic deactivation |
| 78 | blaL1 | CACCGGGTTACCAGCTGAAG | GCGAAGCTGCGCTTGTAGTC | *blaL1* | Beta lactam | antibiotic deactivation |
| 79 | blaMOX/blaCMY | CTATGTCAATGTGCCGAAGCA | GGCTTGTCCTCTTTCGAATAGC | *blaMOX/blaCMY* | Beta lactam | antibiotic deactivation |
| 80 | blaOCH | GGCGACTTGCGCCGTAT | TTTTCTGCTCGGCCATGAG | *blaOCH* | Beta lactam | antibiotic deactivation |
| 81 | blaOKP | GCCGCCATCACCATGAG | GGTGACGTTGTCACCGATCTG | *blaOKP* | Beta lactam | antibiotic deactivation |
| 82 | blaOXA1/blaOXA30 | CGGATGGTTTGAAGGGTTTATTAT | TCTTGGCTTTTATGCTTGATGTTAA | *blaOXA1/blaOXA30* | Beta lactam | antibiotic deactivation |
| 83 | blaOXA10-01 | CGCAATTATCGGCCTAGAAACT | TTGGCTTTCCGTCCCATTT | *blaOXA10* | Beta lactam | antibiotic deactivation |
| 84 | blaOXA10-02 | CGCAATTATCGGCCTAGAAACT | TTGGCTTTCCGTCCCATTT | *blaOXA10* | Beta lactam | antibiotic deactivation |
| 85 | blaOXA58 | GCAATTGCCTTTTAAACCTGA | CTGCCTTTTCAACAAAACCC | *blaOXA58* | Beta lactam | antibiotic deactivation |
| 86 | blaOXY | CGTTCAGGCGGCAGGTT | GCCGCGATATAAGATTTGAGAATT | *blaOXY* | Beta lactam | antibiotic deactivation |
| 87 | blaPAO | CGCCGTACAACCGGTGAT | GAAGTAATGCGGTTCTCCTTTCA | *blaPAO* | Beta lactam | antibiotic deactivation |
| 88 | blaPSE | TTGTGACCTATTCCCCTGTAATAGAA | TGCGAAGCACGCATCATC | *blaPSE* | Beta lactam | antibiotic deactivation |
| 89 | blaROB | GCAAAGGCATGACGATTGC | CGCGCTGTTGTCGCTAAA | *blaROB* | Beta lactam | antibiotic deactivation |
| 90 | blaSFO | CCGCCGCCATCCAGTA | GGGCCGCCAAGATGCT | *blaSFO* | Beta lactam | antibiotic deactivation |
| 91 | blaSHV-01 | TCCCATGATGAGCACCTTTAAA | TTCGTCACCGGCATCCA | *blaSHV* | Beta lactam | antibiotic deactivation |
| 92 | blaSHV-02 | CTTTCCCATGATGAGCACCTTT | TCCTGCTGGCGATAGTGGAT | *blaSHV* | Beta lactam | antibiotic deactivation |
| 93 | blaSHV-03 | GCGTTATTTTCGCCTGTGTA | AGGTGCTCATCATGGGAAAG | *blaSHV* | Beta lactam | antibiotic deactivation |
| 94 | blaSME | AACGGCTTCATTTTTGTTTAG | GCTTCCGCAATAGTTTTATCA | *blaSME* | Beta lactam | antibiotic deactivation |
| 95 | blaTLA | ACACTTTGCCATTGCTGTTTATGT | TGCAAATTTCGGCAATAATCTTT | *blaTLA* | Beta lactam | antibiotic deactivation |
| 96 | blaVEB | CCCGATGCAAAGCGTTATG | GAAAGATTCCCTTTATCTATCTCAGACAA | *blaVEB* | Beta lactam | antibiotic deactivation |
| 97 | blaVIM | GCACTTCTCGCGGAGATTG | CGACGGTGATGCGTACGTT | *blaVIM* | Beta lactam | antibiotic deactivation |
| 98 | blaZ | GGAGATAAAGTAACAAATCCAGTTAGATATGA | TGCTTAATTTTCCATTTGCGATAAG | *blaZ* | Beta lactam | antibiotic deactivation |
| 99 | carB | GGAGTGAGGCTGACCGTAGAAG | ATCGGCGAAACGCACAAA | *carB* | Macrolide (MLSB) | efflux-pumps |
| 100 | catA1 | GGGTGAGTTTCACCAGTTTTGATT | CACCTTGTCGCCTTGCGTATA | *catA1* | (Flor)/ (Chlor)/ (Am)phenicol | antibiotic deactivation |
| 101 | catB | CACTCGACGCCTTCCAAAG | CCGAGCCTATCCAGACATCATT | *catB* | (Flor)/ (Chlor)/ (Am)phenicol | antibiotic deactivation |
| 102 | catB3 | GCACTCGATGCCTTCCAAAA | AGAGCCGATCCAAACGTCAT | *catB3* | (Flor)/ (Chlor)/ (Am)phenicol | antibiotic deactivation |
| 103 | ceoA | ATCAACACGGACCAGGACAAG | GGAAAGTCCGCTCACGATGA | *ceoA* | Multidrug/ Efflux | efflux-pumps |
| 104 | cepA | AGTTGCGCAGAACAGTCCTCTT | TCGTATCTTGCCCGTCGATAAT | *cepA* | Beta lactam | antibiotic deactivation |
| 105 | cfiA | GCAGCGTTGCTGGACACA | GTTCGGGATAAACGTGGTGACT | *cfiA* | Beta lactam | antibiotic deactivation |
| 106 | cfr | GCAAAATTCAGAGCAAGTTACGAA | AAAATGACTCCCAACCTGCTTTAT | *cfr* | (Flor)/ (Chlor)/ (Am)phenicol | antibiotic deactivation |
| 107 | cfxA | TCATTCCTCGTTCAAGTTTTCAGA | TGCAGCACCAAGAGGAGATGT | *cfxA* | Beta lactam | antibiotic deactivation |
| 108 | cmeA | GCAGCAAAGAAGAAGCACCAA | AGCAGGGTAAGTAAAACTAAGTGGTAAATCT | *cmeA* | Multidrug/ Efflux | efflux-pumps |
| 109 | cmlA1-01 | TAGGAAGCATCGGAACGTTGAT | CAGACCGAGCACGACTGTTG | *cmlA1* | (Flor)/ (Chlor)/ (Am)phenicol | efflux-pumps |
| 110 | cmlA1-02 | AGGAAGCATCGGAACGTTGA | ACAGACCGAGCACGACTGTTG | *cmlA1* | (Flor)/ (Chlor)/ (Am)phenicol | efflux-pumps |
| 111 | cmlA5 | GCGCTCTTCGAGGATTCG | CCGCCCAAGCAGAAGTAGAC | *cmlA5* | (Flor)/ (Chlor)/ (Am)phenicol | efflux-pumps |
| 112 | cmr | CGGCATCGTCAGTGGAATT | CGGTTCCGAAAAAGATGGAA | *cmr* | Multidrug/ Efflux | efflux-pumps |
| 113 | cmx(A) | GCGATCGCCATCCTCTGT | TCGACACGGAGCCTTGGT | *cmx(A)* | (Flor)/ (Chlor)/ (Am)phenicol | efflux-pumps |
| 114 | cphA | GCGAGCTGCACAAGCTGAT | CGGCCCAGTCGCTCTTC | *cphA* | Beta lactam | antibiotic deactivation |
| 115 | dfrA1-01 | GGAATGGCCCTGATATTCCA | AGTCTTGCGTCCAACCAACAG | *dfrA1* | Trimethoprim | antibiotic deactivation |
| 116 | dfrA1-02 | TTCAGGTGGTGGGGAGATATAC | TTAGAGGCGAAGTCTTGGGTAA | *dfrA1* | Trimethoprim | antibiotic deactivation |
| 117 | dfrA12 | CCTCTACCGAACCGTCACACA | GCGACAGCGTTGAAACAACTAC | *dfrA12* | Trimethoprim | antibiotic deactivation |
| 118 | disul | TCATCTGCCAAACTCGTCGTTA | GTCAAAGAACGCCGCAATGT | *disul* | Other (Housekeeping) | other (housekeeping) |
| 119 | emrB-01 | CTTTTCTCTAACCGTACATTATCTACGATAAA | AGAACGTAGCGACTGATAAAATGCT | *emrB* | Multidrug/ Efflux | efflux-pumps |
| 120 | emrB-02 | GCAGTAGAAGGAACGATTGTTAGTACAG | TGCGTAAACCCAGCTAACAAGTT | *emrB* | Multidrug/ Efflux | efflux-pumps |
| 121 | emrD | CTCAGCAGTATGGTGGTAAGCATT | ACCAGGCGCCGAAGAAC | *emrD* | Multidrug/ Efflux | efflux-pumps |
| 122 | ereA | CCTGTGGTACGGAGAATTCATGT | ACCGCATTCGCTTTGCTT | *ereA* | Macrolide (MLSB) | antibiotic deactivation |
| 123 | ereB | GCTTTATTTCAGGAGGCGGAAT | TTTTAAATGCCACAGCACAGAATC | *ereB* | Macrolide (MLSB) | antibiotic deactivation |
| 124 | erm(34) | GCGCGTTGACGACGATTT | TGGTCATACTCGACGGCTAGAAC | *erm(34)* | Macrolide (MLSB) | cellular protection |
| 125 | erm(35) | TTGAAAACGATGTTGCATTAAGTCA | TCTATAATCACAACTAACCACTTGAACGT | *erm(35)* | Macrolide (MLSB) | cellular protection |
| 126 | erm(36) | GGCGGACCGACTTGCAT | TCTGCGTTGACGACGGTTAC | *erm(36)* | Macrolide (MLSB) | cellular protection |
| 127 | ermA | TTGAGAAGGGATTTGCGAAAAG | ATATCCATCTCCACCATTAATAGTAAACC | *ermA* | Macrolide (MLSB) | cellular protection |
| 128 | ermA/ermTR | ACATTTTACCAAGGAACTTGTGGAA | GTGGCATGACATAAACCTTCATCA | *ermA/ermTR* | Macrolide (MLSB) | cellular protection |
| 129 | ermB | TAAAGGGCATTTAACGACGAAACT | TTTATACCTCTGTTTGTTAGGGAATTGAA | *ermB* | Macrolide (MLSB) | cellular protection |
| 130 | ermC | TTTGAAATCGGCTCAGGAAAA | ATGGTCTATTTCAATGGCAGTTACG | *ermC* | Macrolide (MLSB) | cellular protection |
| 131 | ermF | CAGCTTTGGTTGAACATTTACGAA | AAATTCCTAAAATCACAACCGACAA | *ermF* | Macrolide (MLSB) | cellular protection |
| 132 | ermJ/ermD | GGACTCGGCAATGGTCAGAA | CCCCGAAACGCAATATAATGTT | *ermJ/ermD* | Macrolide (MLSB) | cellular protection |
| 133 | ermK-01 | GTTTGATATTGGCATTGTCAGAGAAA | ACCATTGCCGAGTCCACTTT | *ermK* | Macrolide (MLSB) | cellular protection |
| 134 | ermK-02 | GAGCCGCAAGCCCCTTT | GTGTTTCATTTGACGCGGAGTAA | *ermK* | Macrolide (MLSB) | cellular protection |
| 135 | ermT-01 | GTTCACTAGCACTATTTTTAATGACAGAAGT | GAAGGGTGTCTTTTTAATACAATTAACGA | *ermT* | Macrolide (MLSB) | cellular protection |
| 136 | ermT-02 | GTAAAATCCCTAGAGAATACTTTCATCCA | TGAGTGATATTTTTGAAGGGTGTCTT | *ermT* | Macrolide (MLSB) | cellular protection |
| 137 | ermX | GCTCAGTGGTCCCCATGGT | ATCCCCCCGTCAACGTTT | *ermX* | Macrolide (MLSB) | cellular protection |
| 138 | ermY | TTGTCTTTGAAAGTGAAGCAACAGT | TAACGCTAGAGAACGATTTGTATTGAG | *ermY* | Macrolide (MLSB) | cellular protection |
| 139 | fabK | TTTCAGCTCAGCACTTTGGTCAT | AAGGCATCTTTTTCAGCCAGTTC | *fabK* | Other^2^ | other |
| 140 | floR-01 | ATTGTCTTCACGGTGTCCGTTA | CCGCGATGTCGTCGAACT | *floR* | (Flor)/ (Chlor)/ (Am)phenicol | efflux-pumps |
| 141 | floR-02 | TCGTCATCTACGGCCTTTTC | TCGTCATCTACGGCCTTTTC | *floR* | (Flor)/ (Chlor)/ (Am)phenicol | efflux-pumps |
| 142 | folA | CGAGCAGTTCCTGCCAAAG | CCCAGTCATCCGGTTCATAATC | *folA* | Trimethoprim | antibiotic deactivation |
| 143 | fosB | TCACTGTAACTAATGAAGCATTAGACCAT | CCATCTGGATCTGTAAAGTAAAGAGATC | *fosB* | Other^3^ | other |
| 144 | fosX | GATTAAGCCATATCACTTTAATTGTGAAAG | TCTCCTTCCATAATGCAAATCCA | *fosX* | Other^3^ | other |
| 145 | fox5 | GGTTTGCCGCTGCAGTTC | GCGGCCAGGTGACCAA | *fox5* | Beta lactam | antibiotic deactivation |
| 146 | gapA | CCGTTGAAGTGAAAGACGGTC | AACCACTTTCTTCGCACCAGC | *gapA* | Other (Housekeeping) | other (housekeeping) |
| 147 | imiR | CCGGACTAGAGCTTCATGTAAGC | CCCACGCGGTACTCTTGTAAA | *imiR* | Multidrug/ Efflux | efflux-pumps |
| 148 | incN_oriT | TTGGGCTTCATAGTACCC | GTGTGATAGCGTGATTTATGC | *oriT-*incN | Other (Plasmid) | other (MGE) |
| 149 | incN_rep | AGTTCACCACCTACTCGCTCCG | CAAGTTCTTCTGTTGGGATTCCG | *rep_*incN | Other (Plasmid) | other (MGE) |
| 150 | incP_oriT | CAGCCTCGCAGAGCAGGAT | CAGCCGGGCAGGATAGGTGAAGT | *oriT-*incP | Other (Plasmid) | other (MGE) |
| 151 | incQ_oriT | TTCGCGCTCGTTGTTCTTCGAGC | GCCGTTAGGCCAGTTTCTCG | *oriT-*incQ | Other (Plasmid) | other (MGE) |
| 152 | incW_trwAB | AGCGTATGAAGCCCGTGAAGGG | AAAGATAAGCGGCAGGACAATAACG | *trwAB* | Other (Plasmid) | other (MGE) |
| 153 | intl1 | GCCTTGATGTTACCCGAGAG | GATCGGTCGAATGCGTGT | *intl1* | Other (Integron) | other (MGE) |
| 154 | intl2-01 | GACGGCTACCCTCTGTTATCTC | GCCACCACTTGTTTGAGGA | *intl2* | Other (Integron) | other (MGE) |
| 155 | intI2-02 | TGCTTTTCCCACCCTTACC | GACGGCTACCCTCTGTTATCTC | *intI2* | Other (Integron) | other (MGE) |
| 156 | intl3-01 | GCCACCACTTGTTTGAGGA | GGATGTCTGTGCCTGCTTG | *intl3* | Other (Integron) | other (MGE) |
| 157 | intI3-02 | GCCACCACTTGTTTGAGGA | GGATGTCTGTGCCTGCTTG | *intI3* | Other (Integron) | other (MGE) |
| 158 | IS1111 | GTCTTAAGGTGGGCTGCGTG | CCCCGAATCTCATTGATCAGC | *IS1111* | Other (Insertion Sequence) | other (MGE) |
| 159 | IS1133 | GCAGCGTCGGGTTGGA | ACGCGTTCGAACAACTGTAATG | *IS1133* | Other (Insertion Sequence) | other (MGE) |
| 160 | IS613 | AGGTTCGGACTCAATGCAACA | TTCAGCACATACCGCCTTGAT | *IS613* | Other (Insertion Sequence) | other (MGE) |
| 161 | ISAba3-Acineto-01 | TCAGAGGCAGCGGTATACGA | GGTTGATTCAGTTAAAGTACGTAAAACTTT | *ISAba3-*Acineto | Other (Insertion Sequence) | other (MGE) |
| 162 | ISAba3-Acineto-02 | TCAGAGGCAGCGGTATACGA | GGTTGATTCAGTTAAAGTACGTAAAACTTT | *ISAba3-*Acineto | Other (Insertion Sequence) | other (MGE) |
| 163 | ISEfm1-Entero | AGGTGTCCATGACGTGAAAGTG | TCCTTTGTCCCCTAGGATATTGG | *ISEfm1-*Entero | Other (Insertion Sequence) | other (MGE) |
| 164 | ISPps1-Pseud | CACACTGCAAAAACGCATCCT | TGTCTTTGGCGTCACAGTTCTC | *ISPps1-*Pseud | Other (Insertion Sequence) | other (MGE) |
| 165 | ISSm2-Xanthob | TGGATCGACCGGTTCCAT | GCTGACCGAGCTGTCCATGT | *ISSm2-*Xanthob | Other (Insertion Sequence) | other (MGE) |
| 166 | lmrA-01 | TTCAGATGCAATGGCGTTTG | ATAATCGGGAACATAATGAGCATAACTAC | *lmrA* | Macrolide (MLSB) | efflux-pumps |
| 167 | lmrA-02 | TCGACGTGACCGTAGTGAACA | CGTGACTACCCAGGTGAGTTGA | *lmrA* | Macrolide (MLSB) | efflux-pumps |
| 168 | lnuA-01 | TGACGCTCAACACACTCAAAAA | TTCATGCTTAAGTTCCATACGTGAA | *lnuA* | Macrolide (MLSB) | antibiotic deactivation |
| 169 | lnuA-02 | AGAATGAAAAAGAAGCTGAGCTTCTT | AAGGTGGCAATTACGTTTTTCAAA | *lnuA* | Macrolide (MLSB) | antibiotic deactivation |
| 170 | lnuB-01 | TGAACATAATCCCCTCGTTTAAAGAT | TAATTGCCCTGTTTCATCGTAAATAA | *lnuB* | Macrolide (MLSB) | antibiotic deactivation |
| 171 | lnuB-02 | AAAGGAGAAGGTGACCAATACTCTGA | GGAGCTACGTCAAACAACCAGTT | *lnuB* | Macrolide (MLSB) | antibiotic deactivation |
| 172 | lnuC | TGGTCAATATAACAGATGTAAACCAGATTT | CACCCCAGCCACCATCAA | *lnuC* | Macrolide (MLSB) | antibiotic deactivation |
| 173 | marR-01 | GCGGCGTACTGGTGAAGCTA | TGCCCTGGTCGTTGATGA | *marR* | Multidrug/ Efflux | efflux-pumps |
| 174 | marR-02 | TCTGGCGTTAGCTTCACCAGTAC | GTGCAAAGGCTGGATCGAA | *marR* | Multidrug/ Efflux | efflux-pumps |
| 175 | marR-03 | GCTGTTGATGACATTGCTCACA | CGGCGTACTGGTGAAGCTAAC | *marR* | Multidrug/ Efflux | efflux-pumps |
| 176 | matA | TAGTAGGCAAGCTCGGTGTTGA | CCTGTGCTATTTTAAGCCTTGTTTCT | *matA* | Macrolide (MLSB) | efflux-pumps |
| 177 | mdetl1 | ATACAGCAGTGGATATTGGTTTAATTGT | TGCATAAGGTGAATGTTCCATGA | *mdetl1* | Multidrug/ Efflux | efflux-pumps |
| 178 | mdh-F_f | AAGAAACGGGCGTACTGACC | GTGGCTGATCTGACCAAACG | *mdh-F_f* | Other (Housekeeping) | other (housekeeping) |
| 179 | mdtA | CCTAACGGGCGTGACTTCA | TTCACCTGTTTCAAGGGTCAAA | *mdtA* | Multidrug/ Efflux | efflux-pumps |
| 180 | mdtE/yhiU | CGTCGGCGCACTCGTT | TCCAGACGTTGTACGGTAACCA | *mdtE/yhiU* | Multidrug/ Efflux | efflux-pumps |
| 181 | mdtF | CCACCATCGGGCTTTCC | CCCTTCTTTCTGCATCATCTCA | *mdtF* | Multidrug/ Efflux | efflux-pumps |
| 182 | mecA | GGTTACGGACAAGGTGAAATACTGAT | TGTCTTTTAATAAGTGAGGTGCGTTAATA | *mecA* | Beta lactam | cellular protection |
| 183 | mefA | CCGTAGCATTGGAACAGCTTTT | AAACGGAGTATAAGAGTGCTGCAA | *mefA* | Macrolide (MLSB) | efflux-pumps |
| 184 | mepA | ATCGGTCGCTCTTCGTTCAC | ATAAATAGGATCGAGCTGCTGGAT | *mepA* | Multidrug/ Efflux | efflux-pumps |
| 185 | merA | GTGCCGTCCAAGATCATG | GGTGGAAGTCCAGTAGGGTGA | *merA* | Other^4^ | other |
| 186 | mexA | AGGACAACGCTATGCAACGAA | CCGGAAAGGGCCGAAAT | *mexA* | Multidrug/ Efflux | efflux-pumps |
| 187 | mexB | CTGGAGATCGACGACGAGAAG | GAAATCGTTGACGTAGCTGGAA | *mexB* | Multidrug/ Efflux | efflux-pumps |
| 188 | mexD | TTGCCACTGGCTTTCATGAG | CACTGCGGAGAACTGTCTGTAGA | *mexD* | Multidrug/ Efflux | efflux-pumps |
| 189 | mexE | GGTCAGCACCGACAAGGTCTAC | AGCTCGACGTACTTGAGGAACAC | *mexE* | (Flor)/ (Chlor)/ (Am)phenicol | efflux-pumps |
| 190 | mexF | CCGCGAGAAGGCCAAGA | TTGAGTTCGGCGGTGATGA | *mexF* | (Flor)/ (Chlor)/ (Am)phenicol | efflux-pumps |
| 191 | mfs | AATTTTGCCGATTATTGCTGAAA | GATTGTCATCATTCGTTTATCACCAA | *mfs* | Multidrug/ Efflux | efflux-pumps |
| 192 | mphA-01 | CTGACGCGCTCCGTGTT | GGTGGTGCATGGCGATCT | *mphA* | Macrolide (MLSB) | antibiotic deactivation |
| 193 | mphA-02 | TGATGACCCTGCCATCGA | TTCGCGAGCCCCTCTTC | *mphA* | Macrolide (MLSB) | antibiotic deactivation |
| 194 | mphB | CGCAGCGCTTGATCTTGTAG | TTACTGCATCCATACGCTGCTT | *mphB* | Macrolide (MLSB) | antibiotic deactivation |
| 195 | mphC | CGTTTGAAGTACCGAATTGGAAA | GCTGCGGGTTTGCCTGTA | *mphC* | Macrolide (MLSB) | antibiotic deactivation |
| 196 | msrA | CTGCTAACACAAGTACGATTCCAAAT | TCAAGTAAAGTTGTCTTACCTACACCATT | *msrA* | Macrolide (MLSB) | efflux-pumps |
| 197 | msrC-01 | TCAGACCGGATCGGTTGTC | CCTATTTTTTGGAGTCTTCTCTCTAATGTT | *msrC* | Macrolide (MLSB) | efflux-pumps |
| 198 | msrC-02 | GAATCACTTGTCCGCAGTTTGTT | CGTACACAACGGTTTCGTCAGA | *msrC* | Macrolide (MLSB) | efflux-pumps |
| 199 | mtrC-01 | GGACGGGAAGATGGTCCAA | CGTAGCGTTCCGGTTCGAT | *mtrC* | Multidrug/ Efflux | efflux-pumps |
| 200 | mtrC-02 | CGGAGTCCATCGACCATTTG | ATCGTCGGCAAGGAGAATCA | *mtrC* | Multidrug/ Efflux | efflux-pumps |
| 201 | mtrD-01 | TGCGCGTAGTCGTTCATCTC | CGTTCCAATTTCCTGATGATTG | *mtrD* | Multidrug/ Efflux | efflux-pumps |
| 202 | mtrD-02 | GGTCGGCACGCTCTTGTC | TGAAGAATTTGCGCACCACTAC | *mtrD* | Multidrug/ Efflux | efflux-pumps |
| 203 | mtrD-03 | CCGCCAAGCCGATATAGACA | GGCCGGGTTGCCAAA | *mtrD* | Multidrug/ Efflux | efflux-pumps |
| 204 | ndm | GGCCACACCAGTGACAATATCA | CAGGCAGCCACCAAAAGC | *ndm* | Beta lactam | antibiotic deactivation |
| 205 | nimE | TGCGCCAAGATAGGGCATA | GTCGTGAATTCGGCAGGTTTA | *nimE* | Other^5^ | other |
| 206 | nisB-01 | GGGAGAGTTGCCGATGTTGTA | AGCCACTCGTTAAAGGGCAAT | *nisB* | Other^6^ | other |
| 207 | nisB-02 | CGGTTGAACGGCGTGAA | TTTCCACCCAGGTTTGCTACTATT | *nisB* | Other^6^ | other |
| 208 | oleC | CCCGGAGTCGATGTTCGA | GCCGAAGACGTACACGAACAG | *oleC* | Macrolide (MLSB) | efflux-pumps |
| 209 | oprD | ATGAAGTGGAGCGCCATTG | GGCCACGGCGAACTGA | *oprD* | Beta lactam | efflux-pumps |
| 210 | oprJ | ACGAGAGTGGCGTCGACAA | AAGGCGATCTCGTTGAGGAA | *oprJ* | Multidrug/ Efflux | efflux-pumps |
| 211 | orf37-IS26 | GCCGGGTTGTGCAAATAGAC | TGGCAATCTGTCGCTGCTG | *orf37*-IS26 | Other (Insertion Sequence) | other (MGE) |
| 212 | orf39-IS26 | GCGCGTCGAGCATCAATAG | CAGTTGTGCTGCTGGTGGTC | *orf39*-IS26 | Other (Insertion Sequence) | other (MGE) |
| 213 | pAKD1-IncP-1β | GGTAAGATTACCGATAAACT | GTTCGTGAAGAAGATGTA | *pAKD1-*IncP-1β | Other (Plasmid) | other (MGE) |
| 214 | pAMBL-1-F | CAGGCTCTTAATGTGATA | TTATGCTCAATACTCGTG | *pAMBL-1-F* | Other (Plasmid) | other (MGE) |
| 215 | pbp | CCGGTGCCATTGGTTTAGA | AAAATAGCCGCCCCAAGATT | *pbp* | Beta lactam | cellular protection |
| 216 | pbp2b | AGACGGTAACGTATAACTTTTTGAAAGA | GCGTGTAGCCGGCAATG | *pbp2b* | Beta lactam | cellular protection |
| 217 | pbp2x | TTTCATAAGTATCTGGACATGGAAGAA | CCAAAGGAAACTTGCTTGAGATTAG | *pbp2x* | Beta lactam | cellular protection |
| 218 | pbp5 | GGCGAACTTCTAATTAATCCTATCCA | CGCCGATGACATTCTTCTTATCTT | *pbp5* | Beta lactam | cellular protection |
| 219 | pBS228-IncP-1α | CAATCCATCGACAATCAC | GACAATCAGCTACTTCAC | *pBS228-*IncP-1α | Other (Plasmid) | other (MGE) |
| 220 | penA | AGACGGTAACGTATAACTTTTTGAAAGA | GCGTGTAGCCGGCAATG | *penA* | Beta lactam | cellular protection |
| 221 | pikR1 | TCGACATGCGTGACGAGATT | CCGCGAATTAGGCCAGAA | *pikR1* | Macrolide (MLSB) | cellular protection |
| 222 | pikR2 | TCGTGGGCCAGGTGAAGA | TTCCCCTTGCCGGTGAA | *pikR2* | Macrolide (MLSB) | cellular protection |
| 223 | pmrA | TTTGCAGGTTTTGTTCCTAATGC | GCAGAGCCTGATTTCTCCTTTG | *pmrA* | Multidrug/ Efflux | efflux-pumps |
| 224 | pncA | GCAATCGAGGCGGTGTTC | TTGCCGCAGCCAATTCA | *pncA* | Other^7^ | other |
| 225 | pNI105-F | CGCTAAGGATGTTTACAC | CTCAACCGTTCTAGGATT | *pNI105-F* | Other (Plasmid) | other (MGE) |
| 226 | pNI105map-F | CCCCCAGGACTTGCGAGCG | GAGGCATGCACGCCGACCA | *pNI105map-F* | Other (Plasmid) | other (MGE) |
| 227 | qac-01 | CAATAATAACCGAAATAATAGGGACAAGTT | AATAAGTGTTCCTAGTGTTGGCCATAG | *qac* | Other^8^ | other |
| 228 | qac-02 | GGAGATTTAGCTCATGTAGCTGAAGAA | AAGCTGTTTTATCCCCGTAGCTTTA | *qac* | Other^8^ | other |
| 229 | qacA | TGGCAATAGGAGCTATGGTGTTT | AAGGTAACACTATTTTCGGTCCAAATC | *qacA* | Other^8^ | other |
| 230 | qacA/qacB | TTTAGGCAGCCTCGCTTCA | CCGAATCCAAATAAAACCCAATAA | *qacA/qacB* | Other^8^ | other |
| 231 | qacE∆1-01 | TCGCAACATCCGCATTAAAA | ATGGATTTCAGAACCAGAGAAAGAAA | *qacE∆1* | Other^8^ | other |
| 232 | qacE∆1-02 | CCCCTTCCGCCGTTGT | CGACCAGACTGCATAAGCAACA | *qacE∆1* | Other^8^ | other |
| 233 | qacH-01 | GTGGCAGCTATCGCTTGGAT | CCAACGAACGCCCACAA | *qacH* | Other^8^ | other |
| 234 | qacH-02 | CATCGTGCTTGTGGCAGCTA | TGAACGCCCAGAAGTCTAGTTTT | *qacH* | Other^8^ | other |
| 235 | qacH-03 | GTCGGTGTTGCTTATGCAGTCT | CAACCAGGCAATGGCTGTAA | *qacH* | Other^8^ | other |
| 236 | qnrA | AGGATTTCTCACGCCAGGATT | CCGCTTTCAATGAAACTGCAA | *qnrA* | (Flor)/ (Chlor)/ (Am)phenicol | cellular protection |
| 237 | qnrB | GCGACGTTCAGTGGTTCAGA | GCTGCTCGCCAGTCGAA | *qnrB* | (Flor)/ (Chlor)/ (Am)phenicol | cellular protection |
| 238 | rarD-01 | GCGGGTGTGGTCACTACGAT | AGCGTTGGGCCGATATACTG | *rarD* | (Flor)/ (Chlor)/ (Am)phenicol | efflux-pumps |
| 239 | rarD-02 | TGACGCATCGCGTGATCT | AAATTTTCTGTGGCGTCTGAATC | *rarD* | (Flor)/ (Chlor)/ (Am)phenicol | efflux-pumps |
| 240 | rpoB | CGAACATCGGTCTGATCAACTC | GTTGCATGTTCGCACCCAT | *rpoB* | Other (Housekeeping) | other (housekeeping) |
| 241 | sat4 | GAATGGGCAAAGCATAAAAACTTG | CCGATTTTGAAACCACAATTATGATA | *sat4* | Other^9^ | other |
| 242 | sdeB-01 | CACTACCGCTTCCGCACTTAA | TGAAAAAACGGGAAAAGTCCAT | *sdeB* | Multidrug/ Efflux | efflux-pumps |
| 243 | sdeB-02 | GGCATGCAGAAAGTGTTTATGC | TTAAGTGCGGAAGCGGTAGTG | *sdeB* | Multidrug/ Efflux | efflux-pumps |
| 244 | spcN-01 | AAAAGTTCGATGAAACACGCCTAT | TCCAGTGGTAGTCCCCGAATC | *spcN* | Aminoglycoside | antibiotic deactivation |
| 245 | spcN-02 | CAGAATCTTCCTGAAAAGTTTGATGAA | CGCAGACACGCCGAATC | *spcN* | Aminoglycoside | antibiotic deactivation |
| 246 | speA | GCAAGAGGTATTTGCTCAACAAGA | CAGGGTCACCCTCATAAAGAAAA | *speA* | Other^10^ | other |
| 247 | str | AATGAGTTTTGGAGTGTCTCAACGTA | AATCAAAACCCCTATTAAAGCCAAT | *str* | Aminoglycoside | antibiotic deactivation |
| 248 | strA | CCGGTGGCATTTGAGAAAAA | GTGGCTCAACCTGCGAAAAG | *strA* | Aminoglycoside | antibiotic deactivation |
| 249 | strB | GCTCGGTCGTGAGAACAATCT | CAATTTCGGTCGCCTGGTAGT | *strB* | Aminoglycoside | antibiotic deactivation |
| 250 | sul1-01 | CAGCGCTATGCGCTCAAG | ATCCCGCTGCGCTGAGT | *sul1* | Sulfonamide | cellular protection |
| 251 | sul1-02 | TCCGATGGAGGCCGGTATCTGG | CGGGAATGCCATCTGCCTTGAG | *sul1* | Sulfonamide | cellular protection |
| 252 | sul1-03 | GCCGATGAGATCAGACGTATTG | CGCATAGCGCTGGGTTTC | *sul1* | Sulfonamide | cellular protection |
| 253 | sulA/folP-01 | CAGGCTCGTAAATTGATAGCAGAAG | CTTTCCTTGCGAATCGCTTT | *sulA/folP* | Sulfonamide | cellular protection |
| 254 | sulA/folP-02 | GCGATTCGCAAGGAAAGTGA | CACATGGGCCATTTTTTCATC | *sulA/folP* | Sulfonamide | cellular protection |
| 255 | sulA/folP-03 | CACGGCTTCGGCTCATGT | TGCCATCCTGTGACTAGCTACGT | *sulA/folP* | Sulfonamide | cellular protection |
| 256 | tet(32) | CCATTACTTCGGACAACGGTAGA | CAATCTCTGTGAGGGCATTTAACA | *tet(32)* | Tetracycline | cellular protection |
| 257 | tet(35) | ACCCCATGACGTACCTGTAGAGA | CAACCCACACTGGCTACCAGTT | *tet(35)* | Tetracycline | efflux-pumps |
| 258 | tet(36)-01 | AGAATACTCAGCAGAGGTCAGTTCCT | TGGTAGGTCGATAACCCGAAAAT | *tet(36)* | Tetracycline | cellular protection |
| 259 | tet(36)-02 | TGCAGGAAAGACCTCCATTACAG | CTTTGTCCACACTTCCACGTACTATG | *tet(36)* | Tetracycline | cellular protection |
| 260 | tet(37) | GAGAACGTTGAAAAGGTGGTGAA | AACCAAGCCTGGATCAGTCTCA | *tet(37)* | Tetracycline | antibiotic deactivation |
| 261 | tet(38) | TTAATGTGGCGGTATCTGTAGGTATT | TTGCCTGGGAAATTTAATGCTTT | *tet(38)* | Tetracycline | efflux-pumps |
| 262 | tetA-01 | GCTGTTTGTTCTGCCGGAAA | GGTTAAGTTCCTTGAACGCAAACT | *tetA* | Tetracycline | efflux-pumps |
| 263 | tetA-02 | CTCACCAGCCTGACCTCGAT | CACGTTGTTATAGAAGCCGCATAG | *tetA* | Tetracycline | efflux-pumps |
| 264 | tetB-01 | AGTGCGCTTTGGATGCTGTA | AGCCCCAGTAGCTCCTGTGA | *tetB* | Tetracycline | efflux-pumps |
| 265 | tetB-02 | GCCCAGTGCTGTTGTTGTCAT | TGAAAGCAAACGGCCTAAATACA | *tetB* | Tetracycline | efflux-pumps |
| 266 | tetC-01 | CATATCGCAATACATGCGAAAAA | AAAGCCGCGGTAAATAGCAA | *tetC* | Tetracycline | efflux-pumps |
| 267 | tetC-02 | ACTGGTAAGGTAAACGCCATTGTC | ATGCATAAACCAGCCATTGAGTAAG | *tetC* | Tetracycline | efflux-pumps |
| 268 | tetC-03 | TGCGTTGATGCAATTTCTATGC | GGAATGGTGCATGCAAGGAG | *tetC* | Tetracycline | efflux-pumps |
| 269 | tetD-01 | TGCCGCGTTTGATTACACA | CACCAGTGATCCCGGAGATAA | *tetD* | Tetracycline | efflux-pumps |
| 270 | tetD-02 | TGTCATCGCGCTGGTGATT | CATCCGCTTCCGGGAGAT | *tetD* | Tetracycline | efflux-pumps |
| 271 | tetD-03 | CTGGACGCGATGGGAATT | TCCGCTTCCGGGAGATATT | *tetD* | Tetracycline | efflux-pumps |
| 272 | tetE | TTGGCGCTGTATGCAATGAT | CGACGACCTATGCGATCTGA | *tetE* | Tetracycline | efflux-pumps |
| 273 | tetG-01 | TCAACCATTGCCGATTCGA | TGGCCCGGCAATCATG | *tetG* | Tetracycline | efflux-pumps |
| 274 | tetG-02 | CATCAGCGCCGGTCTTATG | CCCCATGTAGCCGAACCA | *tetG* | Tetracycline | efflux-pumps |
| 275 | tetH | TTTGGGTCATCTTACCAGCATTAA | TTGCGCATTATCATCGACAGA | *tetH* | Tetracycline | efflux-pumps |
| 276 | tetJ | GGGTGCCGCATTAGATTACCT | TCGTCCAATGTAGAGCATCCATA | *tetJ* | Tetracycline | efflux-pumps |
| 277 | tetK | CAGCAGTCATTGGAAAATTATCTGATTATA | CCTTGTACTAACCTACCAAAAATCAAAATA | *tetK* | Tetracycline | efflux-pumps |
| 278 | tetL-01 | AGCCCGATTTATTCAAGGAATTG | CAAATGCTTTCCCCCTGTTCT | *tetL* | Tetracycline | efflux-pumps |
| 279 | tetL-02 | ATGGTTGTAGTTGCGCGCTATAT | ATCGCTGGACCGACTCCTT | *tetL* | Tetracycline | efflux-pumps |
| 280 | tetM-01 | CATCATAGACACGCCAGGACATAT | CGCCATCTTTTGCAGAAATCA | *tetM* | Tetracycline | cellular protection |
| 281 | tetM-02 | TAATATTGGAGTTTTAGCTCATGTTGATG | CCTCTCTGACGTTCTAAAAGCGTATTAT | *tetM* | Tetracycline | cellular protection |
| 282 | tetM-03 | GCAATTCTACTGATTTCTGC | CTGTTTGATTACAATTTCCGC | *tetM* | Tetracycline | cellular protection |
| 283 | tetO-01 | ATGTGGATACTACAACGCATGAGATT | TGCCTCCACATGATATTTTTCCT | *tetO* | Tetracycline | cellular protection |
| 284 | tetO-02 | CAACATTAACGGAAAGTTTATTGTATACCA | TTGACGCTCCAAATTCATTGTATC | *tetO* | Tetracycline | cellular protection |
| 285 | tetPA | AGTTGCAGATGTGTATAGTCGTAAACTATCTATT | TGCTACAAGTACGAAAACAAAACTAGAA | *tetPA* | Tetracycline | efflux-pumps |
| 286 | tetPB-01 | ACACCTGGACACGCTGATTTT | ACCGTCTAGAACGCGGAATG | *tetPB* | Tetracycline | cellular protection |
| 287 | tetPB-02 | TGATACACCTGGACACGCTGAT | CGTCCAAAACGCGGAATG | *tetPB* | Tetracycline | cellular protection |
| 288 | tetPB-03 | TGGGCGACAGTAGGCTTAGAA | TGACCCTACTGAAACATTAGAAATATACCT | *tetPB* | Tetracycline | cellular protection |
| 289 | tetPB-04 | AGTGGTGCAAATACTGAAAAAGTTGT | TTTGTTCCTTCGTTTTGGACAGA | *tetPB* | Tetracycline | cellular protection |
| 290 | tetPB-05 | CTGAAGTGGAGCGATCATTCC | CCCTCAACGGCAGAAATAACTAA | *tetPB* | Tetracycline | cellular protection |
| 291 | tetQ | CGCCTCAGAAGTAAGTTCATACACTAAG | TCGTTCATGCGGATATTATCAGAAT | *tetQ* | Tetracycline | cellular protection |
| 292 | tetR-01 | ATGAGTTCGGCCAGAATTTCC | GGTTGTGCGCGAAATGATT | *tetR* | Tetracycline | efflux-pumps |
| 293 | tetR-02 | CGCGATAGACGCCTTCGA | TCCTGACAACGAGCCTCCTT | *tetR* | Tetracycline | efflux-pumps |
| 294 | tetR-03 | CGCGATGGAGCAAAAGTACAT | AGTGAAAAACCTTGTTGGCATAAAA | *tetR* | Tetracycline | efflux-pumps |
| 295 | tetS | TTAAGGACAAACTTTCTGACGACATC | TGTCTCCCATTGTTCTGGTTCA | *tetS* | Tetracycline | cellular protection |
| 296 | tetT | CCATATAGAGGTTCCACCAAATCC | TGACCCTATTGGTAGTGGTTCTATTG | *tetT* | Tetracycline | cellular protection |
| 297 | tetU-01 | GTGGCAAAGCAACGGATTG | TGCGGGCTTGCAAAACTATC | *tetU* | Tetracycline | antibiotic deactivation |
| 298 | tetU-02 | AACAGCGGGTTAAGTGTGCAA | ATGGTATCATTCAGTTTTCCGACAAT | *tetU* | Tetracycline | antibiotic deactivation |
| 299 | tetV | GCGGGAACGACGATGTATATC | CCGCTATCTCACGACCATGAT | *tetV* | Tetracycline | efflux-pumps |
| 300 | tetW | ATGAACATTCCCACCGTTATCTTT | ATATCGGCGGAGAGCTTATCC | *tetW* | Tetracycline | cellular protection |
| 301 | tetX | AAATTTGTTACCGACACGGAAGTT | CATAGCTGAAAAAATCCAGGACAGTT | *tetX* | Tetracycline | antibiotic deactivation |
| 302 | tnp614 | GGAAATCAACGGCATCCAGTT | CATCCATGCGCTTTTGTCTCT | *tnp614* | Other (Transposon) | other (MGE) |
| 303 | tnpA-01/ Tn21 | CATCATCGGACGGACAGAATT | GTCGGAGATGTGGGTGTAGAAAGT | *Tn21* | Other (Transposon) | other (MGE) |
| 304 | tnpA-02/ IS4 | GGGCGGGTCGATTGAAA | GTGGGCGGGATCTGCTT | *IS4* | Other (Transposon) | other (MGE) |
| 305 | tnpA-03/ IS6 | AATTGATGCGGACGGCTTAA | TCACCAAACTGTTTATGGAGTCGTT | *IS6* | Other (Transposon) | other (MGE) |
| 306 | tnpA-04/ IS6100 | CCGATCACGGAAAGCTCAAG | GGCTCGCATGACTTCGAATC | *IS6100* | Other (Transposon) | other (MGE) |
| 307 | tnpA-05/ IS26 | GCCGCACTGTCGATTTTTATC | GCGGGATCTGCCACTTCTT | *IS26* | Other (Transposon) | other (MGE) |
| 308 | tnpA-06/ IS1216 | TGCAGATGGTTTAACCTTGGATATTT | TCGGTTCATCAAACTGCTTCAC | *IS1216* | Other (Transposon) | other (MGE) |
| 309 | tnpA-07/ ISEcp1 | GAAACCGATGCTACAATATCCAATTT | CAGCACCGTTTGCAGTGTAAG | *ISEcp1* | Other (Transposon) | other (MGE) |
| 310 | tolC-01 | GGCCGAGAACCTGATGCA | AGACTTACGCAATTCCGGGTTA | *tolC* | Multidrug/ Efflux | efflux-pumps |
| 311 | tolC-02 | CAGGCAGAGAACCTGATGCA | CGCAATTCCGGGTTGCT | *tolC* | Multidrug/ Efflux | efflux-pumps |
| 312 | tolC-03 | GCCAGGCAGAGAACCTGATG | CGCAATTCCGGGTTGCT | *tolC* | Multidrug/ Efflux | efflux-pumps |
| 313 | trfA | ACGAAGAAATGGTTGTCCTGTTC | CGTCAGCTTGCGGTACTTCTC | *trfA* | Other (Plasmid) | other (MGE) |
| 314 | ttgA | ACGCCAATGCCAAACGATT | GTCACGGCGCAGCTTGA | *ttgA* | Multidrug/ Efflux | efflux-pumps |
| 315 | uidA | AACCACGCGTCTGTTGACTG | CCCGGTTGCCAGAGGTG | *uidA* | Other (Housekeeping) | other (housekeeping) |
| 316 | vanA | AAAAGGCTCTGAAAACGCAGTTAT | CGGCCGTTATCTTGTAAAAACAT | *vanA* | Vancomycin | cellular protection |
| 317 | vanB-01 | TTGTCGGCGAAGTGGATCA | AGCCTTTTTCCGGCTCGTT | *vanB* | Vancomycin | cellular protection |
| 318 | vanB-02 | CCGGTCGAGGAACGAAATC | TCCTCCTGCAAAAAAAGATCAAC | *vanB* | Vancomycin | cellular protection |
| 319 | vanB-03 | GGCTGTTTCGGGCTGTGA | AACAACTAACGCGGCACTGTT | *vanB* | Vancomycin | cellular protection |
| 320 | vanC-01 | ACAGGGATTGGCTATGAACCAT | TGACTGGCGATGATTTGACTATG | *vanC* | Vancomycin | cellular protection |
| 321 | vanC-02 | CCTGCCACAATCGATCGTT | CGGCTTCATTCGGCTTGATA | *vanC* | Vancomycin | cellular protection |
| 322 | vanC-03 | AAATCAATACTATGCCGGGCTTT | CCGACCGCTGCCATCA | *vanC* | Vancomycin | cellular protection |
| 323 | vanC1 | AGGCGATAGCGGGTATTGAA | CAATCGTCAATTGCTCATTTCC | *vanC1* | Vancomycin | cellular protection |
| 324 | vanC2/vanC3 | TTTGACTGTCGGTGCTTGTGA | TCAATCGTTTCAGGCAATGG | *vanC2/vanC3* | Vancomycin | cellular protection |
| 325 | vanD | CAGAGGAACATAATGTTTCGATAAAATCT | GCCGGATTTTGTGATTCCAA | *vanD* | Vancomycin | cellular protection |
| 326 | vanG | ATTTGAATTGGCAGGTATACAGGTTA | TGATTTGTCTTTGTCCATACATAATGC | *vanG* | Vancomycin | cellular protection |
| 327 | vanHB | GAGGTTTCCGAGGCGACAA | CTCTCGGCGGCAGTCGTAT | *vanHB* | Vancomycin | cellular protection |
| 328 | vanHD | GTGGCCGATTATACCGTCATG | CGCAGGTCATTCAGGCAAT | *vanHD* | Vancomycin | cellular protection |
| 329 | vanRA-01 | CCCTTACTCCCACCGAGTTTT | TTCGTCGCCCCATATCTCAT | *vanRA* | Vancomycin | cellular protection |
| 330 | vanRA-02 | CCACTCCGGCCTTGTCATT | GCTAACCACATTCCCCTTGTTTT | *vanRA* | Vancomycin | cellular protection |
| 331 | vanRB | GCCCTGTCGGATGACGAA | TTACATAGTCGTCTGCCTCTGCAT | *vanRB* | Vancomycin | cellular protection |
| 332 | vanRC | TGCGGGAAAAACTGAACGA | CCCCCCATACGGTTTTGATTA | *vanRC* | Vancomycin | cellular protection |
| 333 | vanRC4 | AGTGCTTTGGCTTATCTCGAAAA | TCCGGCAGCATCACATCTAA | *vanRC4* | Vancomycin | cellular protection |
| 334 | vanRD | TTATAATGGCAAGGATGCACTAAAGT | CGTCTACATCCGGAAGCATGA | *vanRD* | Vancomycin | cellular protection |
| 335 | vanSA | CGCGTCATGCTTTCAAAATTC | TCCGCAGAAAGCTCAATTTGTT | *vanSA* | Vancomycin | cellular protection |
| 336 | vanSC-01 | ATCAACTGCGGGAGAAAAGTCT | TCCGCTGTTCCGCTTCTT | *vanSC* | Vancomycin | cellular protection |
| 337 | vanSC-02 | GCCATCAGCGAGTCTGATGA | CAGCTGGGATCGTTTTTCCTT | *vanSC* | Vancomycin | cellular protection |
| 338 | vanSE | TGGCCGAAGAAGCAGGAA | CAATAATACTCGTCAAAGGAGTTCTCA | *vanSE* | Vancomycin | cellular protection |
| 339 | vanTC-01 | CACACGCATTTTTTCCCATCTAG | CAGCCAACAGATCATCAAAACAA | *vanTC* | Vancomycin | cellular protection |
| 340 | vanTC-02 | ACAGTTGCCGCTGGTGAAG | CGTGGCTGGTCGATCAAAA | *vanTC* | Vancomycin | cellular protection |
| 341 | vanTE | GTGGTGCCAAGGAAGTTGCT | CGTAGCCACCGCAAAAAAAT | *vanTE* | Vancomycin | cellular protection |
| 342 | vanTG | CGTGTAGCCGTTCCGTTCTT | CGGCATTACAGGTATATCTGGAAA | *vanTG* | Vancomycin | cellular protection |
| 343 | vanWB | CGGACAAAGATACCCCCTATAAAG | AAATAGTAAATTGCTCATCTGGCACAT | *vanWB* | Vancomycin | cellular protection |
| 344 | vanWG | ACATTTTCATTTTGGCAGCTTGTAC | CCGCCATAAGAGCCTACAATCT | *vanWG* | Vancomycin | cellular protection |
| 345 | vanXA | CGCTAAATATGCCACTTGGGATA | TCAAAAGCGATTCAGCCAACT | *vanXA* | Vancomycin | cellular protection |
| 346 | vanXB | AGGCACAAAATCGAAGATGCTT | GGGTATGGCTCATCAATCAACTT | *vanXB* | Vancomycin | cellular protection |
| 347 | vanYB | GGCTAAAGCGGAAGCAGAAA | GATATCCACAGCAAGACCAAGCT | *vanYB* | Vancomycin | cellular protection |
| 348 | vanYD-01 | AAGGCGATACCCTGACTGTCA | ATTGCCGGACGGAAGCA | *vanYD* | Vancomycin | cellular protection |
| 349 | vanYD-02 | CAAACGGAAGAGAGGTCACTTACA | CGGACGGTAATAGGGACTGTTC | *vanYD* | Vancomycin | cellular protection |
| 350 | vatB-01 | GGAAAAAGCAACTCCATCTCTTGA | TCCTGGCATAACAGTAACATTCTGA | *vatB* | Macrolide (MLSB) | antibiotic deactivation |
| 351 | vatB-02 | TTGGGAAAAAGCAACTCCATCT | CAATCCACACATCATTTCCAACA | *vatB* | Macrolide (MLSB) | antibiotic deactivation |
| 352 | vatC-01 | CGGAAATTGGGAACGATGTT | GCAATAATAGCCCCGTTTCCTA | *vatC* | Macrolide (MLSB) | antibiotic deactivation |
| 353 | vatC-02 | CGATGTTTGGATTGGACGAGAT | GCTGCAATAATAGCCCCGTTT | *vatC* | Macrolide (MLSB) | antibiotic deactivation |
| 354 | vatD | TGCAATAGTAGCTGCTAATTCTGTTGTT | TGTTTTATTTCGTTAGCAGGATTTCC | *vatD* | Macrolide (MLSB) | antibiotic deactivation |
| 355 | vatE | GACCGTCCTACCAGGCGTAA | TTGGATTGCCACCGACAATT | *vatE* | Macrolide (MLSB) | antibiotic deactivation |
| 356 | vgaA-01 | CGAGTATTGTGGAAAGCAGCTAGTT | CCCGTACCGTTAGAGCCGATA | *vgaA* | Macrolide (MLSB) | efflux-pumps |
| 357 | vgaA-02 | GACGGGTATTGTGGAAAGCAA | TTTCCTGTACCATTAGATCCGATAATT | *vgaA* | Macrolide (MLSB) | efflux-pumps |
| 358 | vgaB-01 | TAAAAGAGAATAAGGCGCAAGGA | TGTTTAGTAGCATGTTGCATTTTCC | *vgaB* | Macrolide (MLSB) | efflux-pumps |
| 359 | vgb-01 | AGGGAGGGTATCCATGCAGAT | ACCAAATGCGCCCGTTT | *vgb* | Macrolide (MLSB) | antibiotic deactivation |
| 360 | vgb-02 | CCACGATGGCTGCCTTTG | GGCCATGCAGGACGGATAT | *vgb* | Macrolide (MLSB) | antibiotic deactivation |
| 361 | vgbB-01 | CAGCCGGATTCTGGTCCTT | TACGATCTCCATTCAATTGGGTAAA | *vgbB* | Macrolide (MLSB) | antibiotic deactivation |
| 362 | vgbB-02 | ATACGAGCTGCCTAATAAAGGATCTT | TGTGAACCACAGGGCATTATCA | *vgbB* | Macrolide (MLSB) | antibiotic deactivation |
| 363 | yceE/mdtG-01 | TGGCACAAAATATCTGGCAGTT | TTGTGTGGCGATAAGAGCATTAG | *yceE/mdtG* | Multidrug/ Efflux | efflux-pumps |
| 364 | yceE/mdtG-02 | TTATCTGTTTTCTGCTCACCTTCTTTT | GCGTGGTGACAAACAGGCTTA | *yceE/mdtG* | Multidrug/ Efflux | efflux-pumps |
| 365 | yceL/mdtH-01 | TCGGGATGGTGGGCAAT | CGATAACCGAGCCGATGTAGA | *yceL/mdtH* | Multidrug/ Efflux | efflux-pumps |
| 366 | yceL/mdtH-02 | CGCGTGAAACCTTAAGTGCTT | AGACGGCTAAACCCCATATAGCT | *yceL/mdtH* | Multidrug/ Efflux | efflux-pumps |
| 367 | yceL/mdtH-03 | CTGCCGTTAAATGGATGTATGC | ACTCCAGCGGGCGATAGG | *yceL/mdtH* | Multidrug/ Efflux | efflux-pumps |
| 368 | yidY/mdtL-01 | GCAGTTGCATATCGCCTTCTC | CTTCCCGGCAAACAGCAT | *yidY/mdtL* | Multidrug/ Efflux | efflux-pumps |
| 369 | yidY/mdtL-02 | TGCTGATCGGGATTCTGATTG | CAGGCGCGACGAACATAAT | *yidY/mdtL* | Multidrug/ Efflux | efflux-pumps |
| 370 | yyaR | CCGTTGCAAGAAGATTATAGAAAAAA | CAAGCATAAGACCGCATAAATGAT | *yyaR* | Other^9^ | other |

^1^ Bacitracin; ^2^ Tricolsan; ^3^ Fosfomycin; ^4^ Mercury; ^5^ Nitroimidazole; ^6^ Nisin; ^7^ Pyrazinamide; ^8^ Quaternary ammonium compound (antiseptic); ^9^Streptothricin; ^10^ Exotoxin type A

**Table S2.** Gene abundance values relative to the 16S rRNA gene in Rainbow trout raised at the Northern Baltic Sea farms

| **Assay Name** | **Classification of the antibiotics the gene confers resistance to** | **Mechanism of resistance** | **Rainbow trout** | | | | | | | | | | | | | |
| --- | --- | --- | --- | --- | --- | --- | --- | --- | --- | --- | --- | --- | --- | --- | --- | --- |
|  |  |  | **Fecal** | | | | | | | | | | **Skin** | | **Gills** | |
|  |  |  | **small_1** | **small_2** | **small_3** | **small_4** | **small_5** | **big_1** | **big_2** | **big_3** | **big_4** | **big_5** | **small** | **big** | **small** | **big** |
| incN_rep | Other (Plasmid) | other (MGE) |  |  |  |  |  |  |  |  |  |  |  |  |  |  |
| tnpA-01 | Other (Transposon)/ Tn21 | other (MGE) |  |  |  |  |  | 1.E-02 |  |  |  | 5.E-02 |  |  |  |  |
| tnpA-03 | Other (Transposon)/ IS6 | other (MGE) |  | 8.E-02 | 6.E-02 | 5.E-02 | 8.E-02 |  |  | 9.E-03 | 1.E-03 |  |  |  |  |  |
| tnpA-04 | Other (Transposon)/ IS6100 | other (MGE) |  |  |  |  |  | 1.E-02 |  |  |  | 6.E-02 |  |  |  |  |
| tnpA-06 | Other (Transposon)/ IS1216 | other (MGE) | 5.E-01 | 6.E-01 | 7.E-01 | 5.E-01 | 8.E-01 |  |  | 7.E-03 | 4.E-03 |  |  |  |  |  |
| tnpA-07 | Other (Transposon)/ ISEcp1 | other (MGE) |  |  | 4.E-02 | 5.E-02 | 6.E-02 |  |  |  | 4.E-04 |  |  |  |  |  |
| intI1 | Other (Integron) | other (MGE) |  |  |  |  |  | 3.E-02 |  |  |  | 9.E-02 |  |  |  |  |
| qacE∆1-01 | Other (Antiseptic) | other |  |  |  |  |  | 6.E-02 |  |  |  | 2.E-01 |  |  |  |  |
| qacE∆1-02 | Other (Antiseptic) | other |  |  |  |  |  | 4.E-02 |  |  |  | 2.E-01 |  |  |  |  |
| sul1-03 | Sulfonamide | cellular protection |  |  |  |  |  | 3.E-02 |  |  |  | 1.E-01 |  |  |  |  |
| tet(32) | Tetracycline | cellular protection |  |  | 2.E-02 |  |  |  |  |  |  |  |  |  |  |  |
| tetM-01 | Tetracycline | cellular protection |  | 1.E-01 | 1.E-01 | 8.E-02 | 1.E-01 |  |  |  |  |  |  |  |  |  |
| tetM-02 | Tetracycline | cellular protection |  | 1.E-01 | 1.E-01 | 7.E-02 | 1.E-01 |  |  |  |  |  |  |  |  |  |
| tetM-03 | Tetracycline | cellular protection |  |  | 3.E-02 |  |  |  |  |  |  |  |  |  |  |  |
| tetO-01 | Tetracycline | cellular protection |  |  | 2.E-02 |  |  |  |  |  |  |  |  |  |  |  |
| tetO-02 | Tetracycline | cellular protection |  |  | 4.E-02 | 7.E-02 | 5.E-02 |  |  |  |  |  |  |  |  |  |
| tetT | Tetracycline | cellular protection |  |  | 2.E-02 |  |  |  |  |  |  |  |  |  |  |  |
| tetW | Tetracycline | cellular protection |  |  | 3.E-02 |  | 5.E-02 |  |  |  |  |  |  |  |  |  |
| dfrA1-01 | Trimethoprim | antibiotic deactivation |  |  |  |  |  | 2.E-02 |  |  |  | 1.E-01 |  |  |  |  |
| drfA1-02 | Trimethoprim | antibiotic deactivation |  |  |  |  |  | 2.E-02 |  |  |  | 6.E-02 |  |  |  |  |
| aadA1 | Aminoglycoside | antibiotic deactivation |  |  |  |  |  | 7.E-03 |  |  |  | 4.E-02 |  |  |  |  |
| aadA2-01 | Aminoglycoside | antibiotic deactivation |  |  |  |  |  | 8.E-03 |  |  |  | 3.E-02 |  |  |  |  |
| aadA2-02 | Aminoglycoside | antibiotic deactivation |  |  |  |  |  | 1.E-02 |  |  |  | 2.E-02 |  |  |  |  |
| aadA2-03 | Aminoglycoside | antibiotic deactivation |  |  |  |  |  | 2.E-02 |  |  |  | 5.E-02 |  |  |  |  |
| catA1 | (Flor)/(Chlor)/(Am)phenicol | antibiotic deactivation |  |  |  |  |  | 9.E-03 |  |  |  | 2.E-02 |  |  |  |  |
| emrB | Multidrug/ Efflux | efflux-pumps |  | 1.E-01 |  |  |  |  | 1.E-02 |  |  | 3.E-03 | 8.E-01 |  |  | 6.E-01 |
| matA | Macrolide (MLSB) | efflux-pumps |  |  | 2.E-02 |  |  |  |  |  |  |  |  |  |  |  |
| mefA | Macrolide (MLSB) | efflux-pumps |  |  | 4.E-02 |  |  |  |  |  |  |  |  |  |  |  |
| msrA | Macrolide (MLSB) | efflux-pumps |  |  |  |  |  |  |  |  |  | 3.E-03 |  |  |  |  |

Missing values: Not detected or C_T_ gene < 27 in the qPCR array

**Table S3.** Gene abundance values relative to the 16S rRNA gene in European whitefish raised at the Northern Baltic Sea farms

| **Assay Name** | **Classification of the antibiotics the gene confers resistance to** | **Mechanism of resistance** | **Whitefish** | | | | | | | | | | | | | |
| --- | --- | --- | --- | --- | --- | --- | --- | --- | --- | --- | --- | --- | --- | --- | --- | --- |
|  |  |  | **Fecal** | | | | | | | | | | **Skin** | | **Gills** | |
|  |  |  | **small_1** | **small_2** | **small_3** | **small_4** | **small_5** | **big_1** | **big_2** | **big_3** | **big_4** | **big_5** | **small** | **big** | **small** | **big** |
| incN_rep | Other (Plasmid) | other (MGE) |  |  |  |  |  |  |  |  |  |  |  | 8.E-01 |  |  |
| tnpA-01 | Other (Transposon)/ Tn21 | other (MGE) |  |  |  |  |  |  |  |  |  |  |  |  |  |  |
| tnpA-03 | Other (Transposon)/ IS6 | other (MGE) | 7.E-02 | 9.E-02 | 6.E-02 |  | 1.E-01 |  |  | 7.E-02 | 8.E-02 |  |  |  |  |  |
| tnpA-04 | Other (Transposon)/ IS6100 | other (MGE) |  |  |  |  |  |  |  |  |  |  |  |  |  |  |
| tnpA-06 | Other (Transposon)/ IS1216 | other (MGE) | 7.E-01 | 9.E-01 | 6.E-01 | 4.E-01 | 6.E-01 | 3.E-01 | 5.E-01 | 6.E-01 | 9.E-01 | 3.E-01 |  |  |  |  |
| tnpA-07 | Other (Transposon)/ ISEcp1 | other (MGE) | 4.E-02 | 4.E-02 | 6.E-02 |  |  |  |  |  | 4.E-02 |  |  |  |  |  |
| intI1 | Other (Integron) | other (MGE) |  |  |  |  |  |  |  |  |  |  |  |  |  |  |
| qacE∆1-01 | Other (Antiseptic) | other |  |  |  |  |  |  |  |  |  |  |  |  |  |  |
| qacE∆1-02 | Other (Antiseptic) | other |  |  |  |  |  |  |  |  |  |  |  |  |  |  |
| sul1-03 | Sulfonamide | cellular protection |  |  |  |  |  |  |  |  |  |  |  |  |  |  |
| tet(32) | Tetracycline | cellular protection | 3.E-02 | 4.E-02 |  |  |  |  |  |  |  |  |  |  |  |  |
| tetM-01 | Tetracycline | cellular protection | 6.E-02 | 1.E-01 | 1.E-01 |  |  |  | 9.E-02 | 2.E-01 | 1.E-01 |  |  |  |  |  |
| tetM-02 | Tetracycline | cellular protection | 6.E-02 | 1.E-01 | 8.E-02 | 7.E-02 |  |  |  | 8.E-02 | 1.E-01 |  |  |  |  |  |
| tetM-03 | Tetracycline | cellular protection |  | 3.E-02 |  |  |  |  |  | 6.E-02 | 5.E-02 |  |  |  |  |  |
| tetO-01 | Tetracycline | cellular protection |  | 1.E-02 |  |  |  |  |  |  | 2.E-02 |  |  |  |  |  |
| tetO-02 | Tetracycline | cellular protection | 5.E-02 | 5.E-02 |  |  |  |  |  |  | 4.E-02 |  |  |  |  |  |
| tetT | Tetracycline | cellular protection |  | 1.E-02 |  |  |  |  |  |  | 2.E-02 |  |  |  |  |  |
| tetW | Tetracycline | cellular protection | 4.E-02 | 3.E-02 |  |  |  |  |  |  | 3.E-02 |  |  |  |  |  |
| dfrA1-01 | Trimethoprim | antibiotic deactivation |  |  |  |  |  |  |  |  |  |  |  |  |  |  |
| drfA1-02 | Trimethoprim | antibiotic deactivation |  |  |  |  |  |  |  |  |  |  |  |  |  |  |
| aadA1 | Aminoglycoside | antibiotic deactivation |  |  |  |  |  |  |  |  |  |  |  |  |  |  |
| aadA2-01 | Aminoglycoside | antibiotic deactivation |  |  |  |  |  |  |  |  |  |  |  |  |  |  |
| aadA2-02 | Aminoglycoside | antibiotic deactivation |  |  |  |  |  |  |  |  |  |  |  |  |  |  |
| aadA2-03 | Aminoglycoside | antibiotic deactivation |  |  |  |  |  |  |  |  |  |  |  |  |  |  |
| catA1 | (Flor)/(Chlor)/(Am)phenicol | antibiotic deactivation |  |  |  |  |  |  |  |  |  |  |  |  |  |  |
| emrB | Multidrug/ Efflux | efflux-pumps | 3.E-02 | 1.E-02 | 7.E-02 |  |  |  |  |  |  |  |  |  |  |  |
| matA | Macrolide (MLSB) | efflux-pumps |  | 3.E-02 |  |  |  |  |  |  | 4.E-02 |  |  |  |  |  |
| mefA | Macrolide (MLSB) | efflux-pumps | 4.E-02 | 5.E-02 |  |  |  |  |  |  | 5.E-02 |  |  |  |  |  |
| msrA | Macrolide (MLSB) | efflux-pumps |  |  |  |  |  |  |  |  |  |  |  |  |  |  |

Missing values: Not detected or C_T_ gene < 27 in the qPCR array

| Table S4. Number of positive qPCR assays and the total assays | | | | | | |
| --- | --- | --- | --- | --- | --- | --- |
| Mechanism of resistance | **Number of positive qPCR assays in fish** | | | | **Number of positive qPCR assays in all samples** | **Total number of qPCR assays** |
|  | Rainbow trout | | Whitefish | |  |  |
| Cellular protection | 9 | | 8 | | 9 | 82 |
| Antibiotic deactivation | 7 | | ND | | 7 | 127 |
| Efflux-pumps | 4 | | 3 | | 4 | 100 |
| Other | 2 | | ND | | 2 | 20 |
| All ARGs | 22 | | 11 | | 22 | 294 |
|  | | | | | | |
| MGEs | **Number of positive qPCR assays in fish** | | | | **Number of positive qPCR assays in all samples** | **Total number of qPCR assays** |
|  | Rainbow trout | | Whitefish | |  |  |
| Transposons | 5 | | 3 | | 5 | 8 |
| Integron | 1 | | ND | | 1 | 5 |
| Plasmid | ND | | 1 | | 1 | 11 |
| Insertion Sequence (IS) | ND | | ND | | ND | 10 |
| All MGEs | 6 | | 4 | | 7 | 34 |
|  | | | | | | |
| Classification of the antibiotics the gene confers resistance to | **Number of positive qPCR assays in fish** | | | | **Number of positive qPCR assays in all samples** | **Total number of qPCR assays** |
|  | Rainbow trout | | Whitefish | |  |  |
| Tetracycline | 8 | | 8 | | 8 | 46 |
| Sulfonamide | 1 | | ND | | 1 | 6 |
| Trimethoprim | 2 | | ND | | 2 | 4 |
| Aminoglycoside | 4 | | ND | | 4 | 36 |
| Macrolide (MSLB) | 3 | | 2 | | 3 | 51 |
| Multidrug/ Efflux | 1 | | 1 | | 1 | 51 |
| (Flor)/(Chlor)/(Am)phenicols | 1 | | ND | | 1 | 32 |
| Other (Antiseptic) | 2 | | ND | | 2 | 20 |
| Beta lactam | ND | | ND | | ND | 62 |
| Vancomycin | ND | | ND | | ND | 34 |
| ND: Not Detected |  |  | |  |  |  |
